# A Design Method of Code Correlation Reference Waveform in GNSS Based on Least-Squares Fitting

**DOI:** 10.3390/s16081194

**Published:** 2016-07-29

**Authors:** Chengtao Xu, Zhe Liu, Xiaomei Tang, Feixue Wang

**Affiliations:** College of Electronic Science and Engineering, National University of Defense Technology, Changsha 410073, China; xct_nudt@163.com (C.X.); 15274999913@163.com (Z.L.); tangxiaomei06@nudt.edu.cn (X.T.)

**Keywords:** GNSS, multipath, BOC, least-squares, SVD, CCRW

## Abstract

The multipath effect is one of the main error sources in the Global Satellite Navigation Systems (GNSSs). The code correlation reference waveform (CCRW) technique is an effective multipath mitigation algorithm for the binary phase shift keying (BPSK) signal. However, it encounters the false lock problem in code tracking, when applied to the binary offset carrier (BOC) signals. A least-squares approximation method of the CCRW design scheme is proposed, utilizing the truncated singular value decomposition method. This algorithm was performed for the BPSK signal, BOC(1,1) signal, BOC(2,1) signal, BOC(6,1) and BOC(7,1) signal. The approximation results of CCRWs were presented. Furthermore, the performances of the approximation results are analyzed in terms of the multipath error envelope and the tracking jitter. The results show that the proposed method can realize coherent and non-coherent CCRW discriminators without false lock points. Generally, there is performance degradation in the tracking jitter, if compared to the CCRW discriminator. However, the performance promotions in the multipath error envelope for the BOC(1,1) and BPSK signals makes the discriminator attractive, and it can be applied to high-order BOC signals.

## 1. Introduction

**Figure 1:**
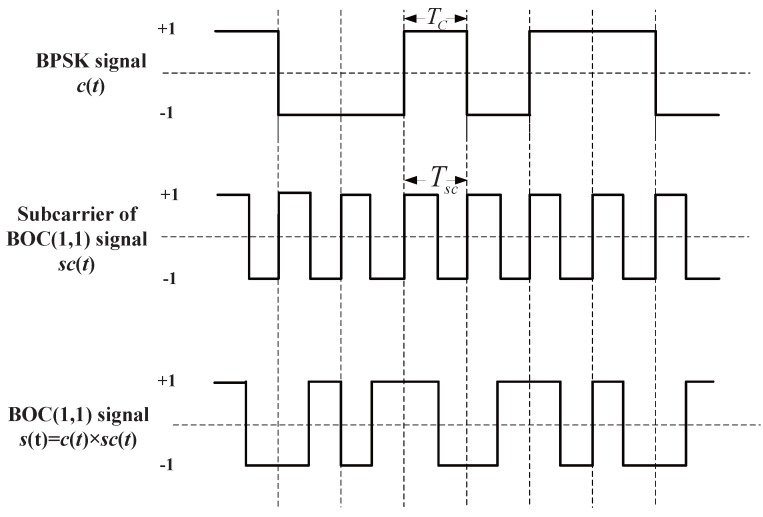
Diagram of the BOC signal and the BPSK signal time series.

The binary offset carrier (BOC) signal, which is adopted by modernized GPS, GLONASS, Galileo and BeiDou satellite navigation systems [1,2,3], is designed for spectrum separation and ranging accuracy improvement. It can be considered as a pseudo-random noise (PRN) code multiplied by a ‘square sine’ subcarrier, which is a sinusoidal carrier with one-bit quantization, as shown in Figure 1. For the BOC(m,n) signal, the code chipping rate is $f_{c}=mf_{0}$, and the frequency of the sine-phased square-wave subcarrier is $f_{sc}=nf_{0},f_{0}=1.023$ MHz. In particular, BOC(1,1) is commonly considered as the interoperability baseline for the modernized BeiDou B1C signal, the Galileo E1 signal and the GPS modernized L1C signal [4,5]. High-order BOC signals, like BOC(10,5), BOC(15,2.5) and BOC(14,2), are designed for providing defense and security services, such as the Galileo Public Regulated Service (PRS), BeiDou military signals and GPS military signals [4,6].

In modern Global Navigation Satellite Systems (GNSSs), the BOC signal is widely used for civil and military applications. These novel signals can improve the tracking accuracy and have better multipath mitigation performance with narrow correlators, when compared to the binary phase shift keying (BPSK) signal. However, two problems emerge in the application of BOC signals, which are the ambiguity in receiver tracking and the lack of multipath mitigation methods [7].

Better ranging accuracy can be achieved, since the BOC signal occupies a larger Gabor bandwidth than the binary phase shift keying (BPSK) signal with the same code frequency. However, the multi-peaked auto-correlation functions (ACF) of BOC signals lead to the possibility of receiver code tracking loop locking onto the side peaks [8], when applying traditional multipath mitigation techniques to the BOC signals.

Many multipath mitigation techniques have been proposed and implemented for the BPSK signal in receiver processing. The narrow correlator technique is primarily proposed in [9] to narrow down the space between correlators in early and late branches. As the code noise variance and multipath error envelope are proportional to the chip spacing, it can enhance the performance of multipath mitigation and tracking precision. The double-delta correlator (ΔΔ) technique is a representative architecture for multipath mitigation techniques using a linear combination of four correlators [10]. By adding another set of early-minus-late correlators beyond the original narrow correlators, it further improves the multipath mitigation performance. The CCRW technique generates a unique local reference waveform instead of local code replicas [11,12], which is distinct from the narrow correlator and double-delta correlator technique. Its resulting cross-correlation function (CCF) of the incoming signal and the local reference waveform is directly a discriminator. By carefully choosing the local code reference waveform, the code multipath mitigation performance can be better, at the expense of the carrier-to-noise ratio (CNR). The W2 waveform in [12] is chosen as the representative CCRW discriminator.

**Figure 2:**
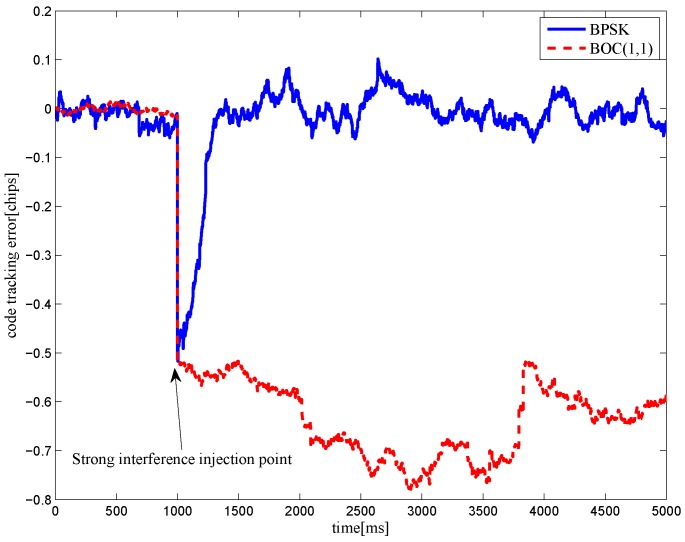
Simulation results of the false lock problem when the CCRW W2 discriminator is applied in a noisy environment (input $C/N_{0}$ = 35 dBHz, injecting a short period of strong interference at a time interval of 1 s, having 0.25 chips of gate width).

**Figure 3:**
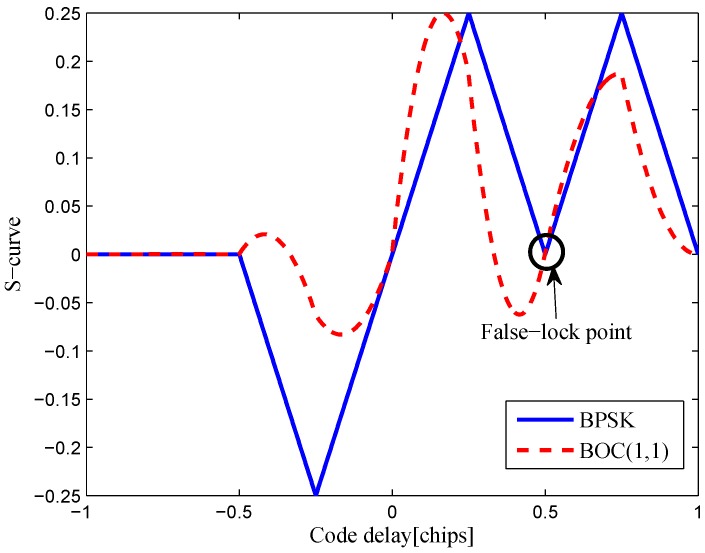
S-curves of non-coherent W2 discriminators (having 0.25 chips of gate width).

The multipath mitigation techniques mentioned above are preferred in the receiver realizations, since they are implemented in the code delay lock loop (DLL) with acceptable hardware cost. However, they are less effective when dealing with BOC signals. Because the resulting DLL discriminator function may have more than one zero-crossing, i.e., they may be tracking on the wrong lock-points and have an intolerable bias in measurements. Furthermore, the filtering effect of the transmission channel is not considered in the CCRW design. Only the zero-crossing in the center of discriminator corresponds to the zero-code-phase error; the other ones would lead to biased pseudo-range measurements. Figure 2 shows the tracking error result for the CCRW W2 technique of BPSK(1) and BOC(1,1) signals. Interference is injected manually at the position of one second, and the tracking error is biased by 0.5 chips accordingly. Thus, the delay lock loop (DLL) of the BOC(1,1) signal would lock into the false steady states and cause biased tracking for the BOC(1,1) signal, but it returned to the correct positions for the BPSK(1) signal. This is due to the false lock point at the position of 0.5 chips of the W2 non-coherent discriminator for the BOC(1,1) signal in Figure 3.

When comparing different multipath mitigation techniques, the common thread can be concluded that they are using locally-generated code replicas in the correlations to shape the code discriminator function (i.e., S-curve). In [12], the optimized S-curve for multipath mitigation is discussed. As a conclusion, the S-curve should return to the zero value at the left side of linear region to restrict the area of the multipath error envelope (MEE).

In this sense, a least-squares approximation was performed by a linear combination of shifted code replicas to approach the ideal S-curve in [12]. Searching the suitable code correlation reference waveform was also performed, like the CCRW technique. However, the standard method of least-squares approximation used in [12] is not capable of handling the ill-conditioned problem brought by the band-limited effect on the coefficient matrix, which is composed of cross-correlation results. The ill-conditioned coefficient matrix could lead to an extremely large condition number in the least-squares approximation, which makes the solution computed with machine precision unreliable. Moreover, the length of the code tracking reference function is not restricted in the simulations, and only the simulations of the BPSK and BOC(n,n) signals coherent discriminator with infinite bandwidth are performed. The work in [13,14] provides an unambiguous non-coherent discriminator for BOC signals using the S-curve shaping method with local linearly-combined codes, based on the least-squares solutions. However, the method has poor tracking precision and requires mass data storage digits.

This paper proposes a least-squares procedure for the code tracking reference function of CCRW, based on the truncated singular value decomposition (SVD) method. By introducing a measurement uncertainty in the low-rank approximation of the coefficient matrix, the condition number could be restrained to less than a preset value. The optimization results of CCRWs are presented and discussed over BPSK, BOC(1,1), BOC(2,1), BOC(6,1) and BOC(7,1) signals in narrow-band situations.

## 2. Least-Squares Method of S-Curve Shaping

The signal distortion by band-limited effects is mainly due to the channel filters in the signal generation and the receiver front-end. The received line-of-sight (LOS) signal in a band-limited receiver can be expressed in a continuous-time model as follows.

(1) $$r\left(t\right)=A\tilde{X}\left(t-\tau_{0}\right)D\left(t-\tau_{0}\right)\cos\left(2\pi f_{0}t+\varphi_{0}\right)+n\left(t\right)$$

Here, r(t) is received with additive white Gaussian noise n(t) of an $N_{0}$ power spectrum density (PSD). *A* is the signal amplitude, and D(t) is the navigation message bit stream, which is ignored in the following. The path delay is given as $\tau_{0}$. The carrier frequency is $f_{0}$, and the carrier phase offset is $\varphi_{0}$. $\tilde{X}\left(t\right)$ is the expression for the baseband BOC waveform X(t) after channel filtering. The baseband waveform X(t)=c(t)·sc(t), where c(t) is the PRN code and $sc\left(t\right)=sign[sin\left(2\pi f_{sc}t\right)]$ is the subcarrier with frequency $f_{sc}$.

(2) $$\begin{array}{ccc} \tilde{X}\left(t\right) & = & X(t)⊗h(t) \end{array}$$

The channel impulse response during the whole signal transmission and receiving is detonated as h(t). Assuming perfect carrier frequency synchronization, the in-phase and quadrature-phase baseband signal after frequency down-conversion are given as follows.

(3) $$\begin{array}{ccc} I(t) & = & A\tilde{X}\left(t-\tau_{0}\right)\cos\left(\varphi_{0}-{\hat{\varphi }}_{0}\right)+n_{I}\left(t\right)\\ Q(t) & = & A\tilde{X}\left(t-\tau_{0}\right)\sin\left(\varphi_{0}-{\hat{\varphi }}_{0}\right)+n_{Q}\left(t\right) \end{array}$$

Here, $\hat{\varphi_{0}}$ is the local estimation of $\varphi_{0}$, $n_{I}\left(t\right)$, and $n_{Q}\left(t\right)$ are the white Gaussian noise of a $N_{0}$ power spectrum density (PSD). For the receiver using the CCRW technique, the reference waveform W(t) is correlated with the baseband signal I(t) and Q(t) with a coherent integration time of *T*. The narrow correlator technique and double-delta technique can be fit into different reference waveforms, as well [7].

(4) $$\begin{array}{ccc} I_{W}\left(\varepsilon \right) & = & AR_{\tilde{X}W}\left(\varepsilon \right)\cos\left(\varphi_{e}\right)+N_{I}\\ Q_{W}\left(\varepsilon \right) & = & AR_{\tilde{X}W}\left(\varepsilon \right)\sin\left(\varphi_{e}\right)+N_{Q} \end{array}$$

**Figure 4:**
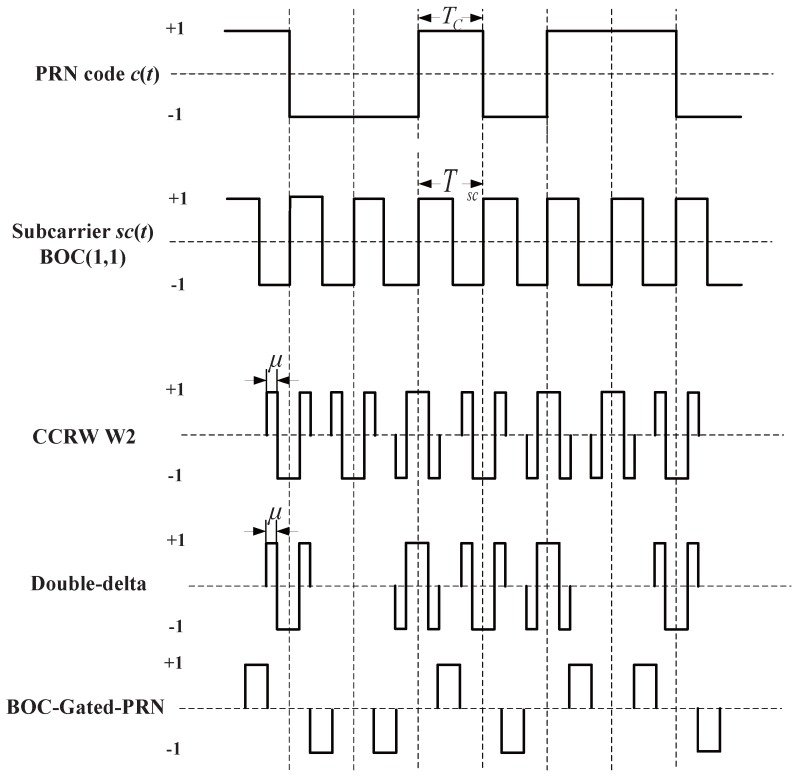
Waveforms of different code correlation reference waveforms.

Here, $\varphi_{e}$ is the estimation error of carrier phase, and *ε* is the code phase estimation error. The correlation result of $\tilde{X}\left(t-\varepsilon \right)$ and W(t) is $R_{\tilde{X}W}\left(\varepsilon \right)$. $N_{I}$ and $N_{Q}$ are the noise part after integration with the variances of $R_{WW}\left(0\right)N_{0}/T$. Basically, there are two types of reference waveforms, i.e., ‘transition-based’ and ‘per-chip’ reference waveforms, as depicted in Figure 4.

For ‘transition-based’ reference waveforms, the reference waveform appears only at the original code transitions. They can be synthesized as a linear combination of multiple instances of time-shifted local code replicas, like the narrow correlator technique and double-delta technique. For ‘per-code’ reference waveforms, the reference waveform appear at a fixed position in each code chip, usually at the chip boundary, like the CCRW W2 technique and the BOC-gated-PRN (BGP) waveform [8]. Here, only the ‘per-code’ reference waveforms are studied, as the ‘transition-based’ reference waveforms were discussed in [12]. The expression of the ‘per-code’ reference waveform can be given as follows.

(5) $$\begin{array}{c} W\left(t\right)=\sum_{j=0}^{\infty }g\left(t-jT_{C}\right)c_{j}\left(t\right) \end{array}$$

Here, g(t) is the element gate, and $c_{j}\left(t\right)$ is the *j*-th PRN code. The element gate occurs at the edge of each chip. By choosing g(t), the S-curve is shaped accordingly. However, the analytic expression of $R_{XW}$ is only resolved for the BPSK signal with infinite bandwidth. Here, a general method for the code tracking reference function design of the BPSK and BOC(m, n) signals with finite bandwidth is proposed.

**Figure 5:**
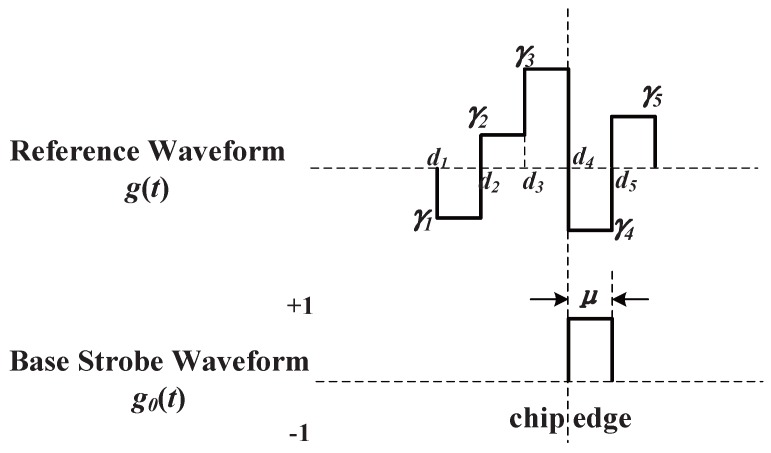
Schematic diagram of the code correlation reference waveform.

W(t) can be described as a combination of series of base strobe waveform $g_{0}\left(t\right)$, as shown in Figure 5.

(6) $$\begin{array}{ccc} W(t) & = & \sum_{i=1}^{N}\gamma_{i}W_{i}\left(t\right)\\ W_{i}\left(t\right) & = & \sum_{j=0}^{\infty }g_{0}\left(t+d_{i}-jT_{c}\right)c_{j}\left(t\right)\\ d_{i} & = & -LT_{c}+\left(i-1\right)\mu \\ W_{0}\left(t\right) & = & \sum_{j=0}^{\infty }g_{0}\left(t-jT_{c}\right)c_{j}\left(t\right) \end{array}$$

Here, $d_{i}$ is the time delay of the *i*-th base strobe waveform, and $\gamma_{i}$ is the weight of the *i*-th base strobe waveform. $W_{0}\left(t\right)$ is a CCRW with the strobe waveform placed at the start of each code chip. In this process, W(t) is manually constructed. Assume $-LT_{c}$ is the initial point of g(t); the base strobe waveform length is *μ*; and *N* is the number of base strobe waveforms in g(t). Notably, Nμ should not exceed the single code chip. The $R_{\tilde{X}W}$ can be considered as a convolution result between $\tilde{X}\left(t\right)$ and $W_{\Sigma }\left(t\right)$.

(7) $$\begin{array}{c} W_{\Sigma }\left(t\right)=\left\{\begin{array}{cc} W(T-t) & 0<t<T\\ 0 & \mathrm{otherwise} \end{array}\right. \end{array}$$

The Fourier transform of $R_{\tilde{X}W}\left(\varepsilon \right)$ can be expressed as a linear combination of time-shifted $R_{\tilde{X}W_{0}}\left(\varepsilon \right)$ filtered by h(t).

(8) $$\begin{array}{ccc} FT\left\{R_{\tilde{X}W}\left(\varepsilon \right)\right\} & = & FT\left\{\tilde{X}\left(t\right)*W_{\Sigma }\left(t\right)\right\}\\ & = & X\left(j\omega \right)H\left(j\omega \right)W_{\Sigma }\left(j\omega \right)\\ & = & H\left(j\omega \right)FT\left\{\sum_{i=1}^{N}\gamma_{i}X\left(t\right)*W_{i}\left(t\right)\right\}\\ & = & H\left(j\omega \right)FT\left\{\sum_{i=1}^{N}\gamma_{i}R_{XW_{0}}\left(\varepsilon +d_{i}\right)\right\} \end{array}$$

Here, FT{x} is the Fourier transform of *x*. The coherent and non-coherent discriminator can be expressed as follows.

(9) $$\begin{array}{ccc} D_{c}\left(\varepsilon \right) & = & R_{\tilde{X}W}\left(\varepsilon \right)\cos\varphi_{e}\\ & = & \sum_{i=1}^{N}\gamma_{i}R_{\tilde{X}W_{0}}\left(\varepsilon +d_{i}\right)\cos\varphi_{e}\\ D_{nc}\left(\varepsilon \right) & = & R_{\tilde{X}X}\left(\varepsilon \right)\sum_{i=1}^{N}\gamma_{i}R_{\tilde{X}W_{0}}\left(\varepsilon +d_{i}\right)\cos\varphi_{e} \end{array}$$

As implied by Equation (9), the S-curve is shaped by setting $\gamma_{i}$ of $W_{i}\left(t\right)$. The ideal S-curve $D_{ideal}\left(\varepsilon \right)$ can be concluded with the following characteristics.

1. Unbiased tracking point, i.e., $D_{ideal}\left(0\right)=0$.
2. Linearity around the tracking point, i.e., $D_{ideal}\left(\varepsilon \right)=\varepsilon$, for $\left|\varepsilon \right|\leq pT_{c}$.
3. Zero value left to the pull-in region, i.e., $D_{ideal}\left(\varepsilon \right)=0$, for $\varepsilon <-pT_{c}$.
4. Avoiding the false lock point, i.e., $D_{ideal}\left(\varepsilon \right)\geq 0$, for ε>0.

Here, $T_{c}$ is the width of the single code chip, and $pT_{c}\left(0<p<1\right)$ is the unilateral range of the linear region. To find the best W(t) that suits the ideal S-curve in band-limited situations, the least-squares method is utilized to fit $D_{ideal}\left(\varepsilon \right)$ by a finite amount of weights, as expressed in Equation (10).

(10) $$\begin{array}{ccc} \gamma & = & \min_{\gamma \in R^{N}}∥D\left(\varepsilon \right)-D_{ideal}\left(\varepsilon \right)∥\\ \varepsilon & = & {\left[-KT_{c},-KT_{c}+\Delta \tau ,-KT_{c}+2\Delta \tau ,...,KT_{c}\right]}^{T} \end{array}$$

Here, $\pm KT_{c}$ is the fitting range of the S-curve for the optimization process, and the length of *ε* is *M*. Combing Equations (9) and (10), the problem can be simplified as finding the solution $x_{LS}$ to Ax=b for the coherent discriminator, and PAx=b for the non-coherent discriminator.

(11) $$\begin{array}{ccc} P & = & diag\left(R_{\tilde{X}X}\left(\varepsilon_{1}\right),R_{\tilde{X}X}\left(\varepsilon_{2}\right),...,R_{\tilde{X}X}\left(\varepsilon_{M}\right)\right)\\ A_{ij} & = & R_{\tilde{X}W_{0}}\left(\varepsilon_{i}+d_{j}\right)\\ b & = & D_{ideal}\left(\varepsilon \right)\\ x & = & {\left[\gamma_{1},\gamma_{2},...,\gamma_{N}\right]}^{T} \end{array}$$

Here, $diag(x_{1},x_{2},...,x_{M})$ stands for the diagonal matrix consisting of $x_{1},x_{2},...,x_{M}$. The coefficient matrix $A\in C^{M\times N}$ is comprised of correlation functions $R_{\tilde{X}W_{0}}$, the observation matrix $b\in R^{M}$ and the unknowns’ vector $x\in R^{N},M\geq N$.

**Figure 6:**
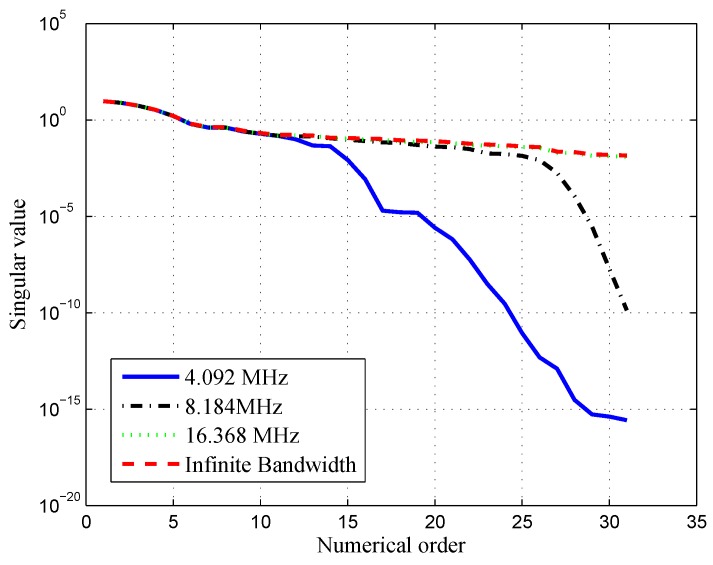
Singular value of the BPSK(1) signal with different bandwidths.

In the band-limited situations, A is often ill-conditioned or rank defected, as can be seen in Figure 6. The coefficient matrix A is ill-conditioned if the condition number κ(A) in Equation (12) is too large. Then, the error mean square for the regression coefficient will largely increase, and as a consequence, the regression fitting will have poor robustness and low precision. That is, for any tiny disturbances δA and δb, the resolution of (A+δA)x=b+δb is distinct from the solution of Ax=b. However, there are always some differences between the coefficient matrix A in the simulations and the actual coefficient matrix A for a real receiver, because of the non-ideal device characteristics or non-ideal channel impulse response. If A is ill-conditioned in the least-squares approximation, the solution would be quite different from the genuine solution. Therefore, the corresponding output in the receiver may not be what is expected. Furthermore, the computational accuracy is limited in the mathematical software; an ill-conditioned problem is probably inaccurate in the software calculation.

(12) $$\begin{array}{ccc} \delta_{x}\left(A\right) & = & \kappa \left(A\right)+\frac{\kappa {\left(A\right)}^{2}\tan\theta }{\eta }\\ \kappa (A) & = & \frac{\sigma_{1}}{\sigma_{r}}\\ \theta & = & \cos^{-1}\frac{∥\mathrm{Ax}∥}{∥b∥}\\ \eta & = & \frac{∥A∥∥x∥}{∥\mathrm{Ax}∥} \end{array}$$

A truncated singular value decomposition (SVD) method is utilized to find $x_{LS}$ in this LS problem and to avoid the ill-conditioned problem of the coefficient matrix.

(13) $$\begin{array}{ccc} {\bar{x}}_{LS} & = & A_{k}^{†}b\\ A_{k}^{†} & = & V_{1}\Sigma_{1}^{-1}U_{1}^{H} \end{array}$$

Here, $A_{k}^{†}$ is the Moore–Penrose (MP) inverse of $A_{k}$, and its SVD is $U_{1}$, $\Sigma_{1}$ and $V_{1}$. The process of the proposed method can be concluded in the following.

Step 1 Do the SVD of $A=U\Sigma V^{H}$, where $\Sigma =diag\left(\sigma_{1},...,\sigma_{r},0,...,0\right),\sigma_{1}\geq ...\geq \sigma_{r}>0$ and rank(A)=r.
Step 2 Set the measurement of uncertainty of A to be $tol=1e^{-5}$.
Step 3 Find $A_{k}=U_{1}\Sigma_{1}V_{1}^{H}$, where $\Sigma_{1}=diag\left(\sigma_{1},...,\sigma_{k}\right),\sigma_{1}\geq ...\geq \sigma_{k}>\sigma_{1}tol>\sigma_{k+1}\geq ...\geq \sigma_{r}$. $U_{1}$ and $V_{1}$ are the first *k* columns of U and V.
Step 4 According to the Equation (13), the approximated minimal norm solution ${\bar{x}}_{LS}$ is acquired.

**Figure 7:**
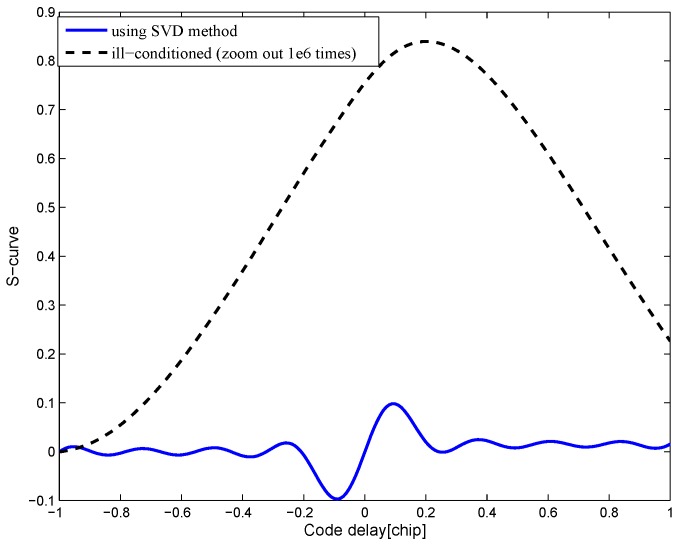
Ill-conditioned S-curves of the BPSK(1) signal with an 8.184-MHz bandwidth.

**Figure 8:**
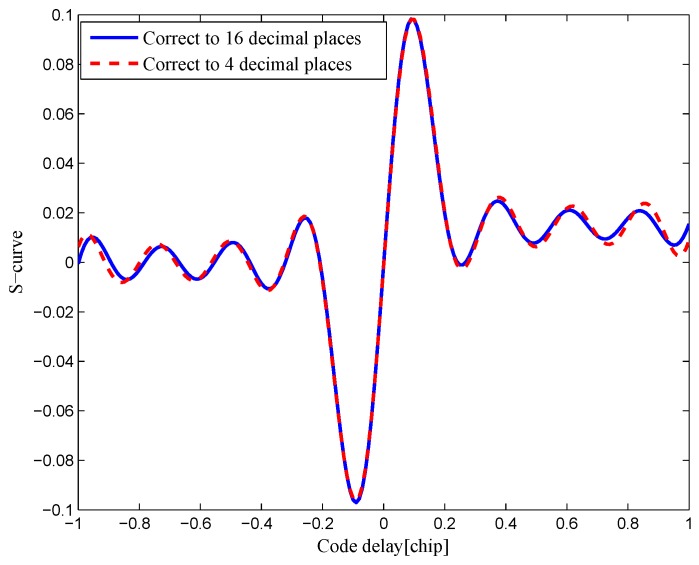
Comparison of the S-curves of the BPSK(1) signal in different precisions with an 8.184-MHz bandwidth.

The measurement of uncertainty tol is employed to exclude the minimal singular value in the ill-conditioned matrix A. The approximate error $∥A-A_{k}∥=\sigma_{k+1}<\sigma_{1}tol$. Take the least-squares solution for the BPSK(1) signal with an 8.184-MHz bandwidth as an example. The resulting S-curve of proposed method is shown in Figure 7, compared to the resulting S-curve acquired by the traditional least-squares solution with the ill-conditioned coefficient matrix. The resulting S-curve of the proposed method can reproduce the target S-curve, but the result in the ill-conditioned case is inaccurate and unusable. Moreover, the proposed S-curves calculated with different data precisions are shown in Figure 8. This shows that there is little difference in the results when the coefficient matrix varies slightly. Therefore, the proposed method can overcome the ill-condition problem.

## 3. Results

The representative results for BPSK(1), BOC(1,1), BOC(2,1), BOC(6,1) and BOC(7,1) signals are presented and discussed in this section. The process for the BPSK(1) signal can be applied to BPSK(n) signals with the same ratio between the chip rate and the bandwidth. Similarly, the results of BOC(1,1), BOC(2,1), BOC(6,1) and BOC(7,1) can be also applied to BOC(n,n), BOC(2n,n), BOC(6n,n) and BOC(7n,n), respectively. The results of CCRW W2, the double-delta correlator, the BGP waveform and the process in [14] are also simulated as for comparisons.

The simulation parameters are listed in Table 1, Table 2, Table 3, Table 4 and Table 5. The fitting range is the simulation range for the S-curve fitting. The CCRWs are constrained within the waveform range (less than one chip), with base strobe waveform gate width of μ. μ is selected according to the bandwidth. If there is a large front-end bandwidth, μ can be a rather small value, since the sampling rate is always greater than the bandwidth. The target S-curve has a linear region around the tracking point and a small offset in the right side to expand the pull-in region and avoid the possible false locking points.

### 3.1. BPSK(1)

**Figure 9:**
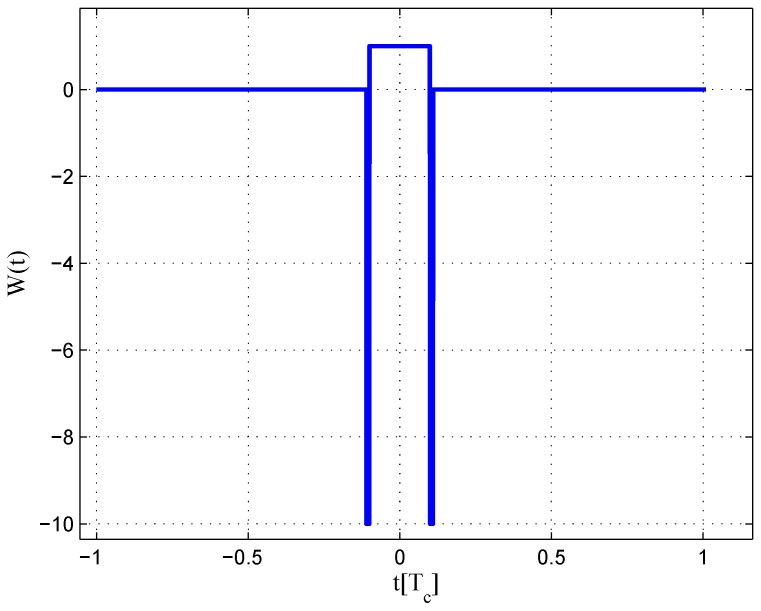
Code correlation reference waveform diagram for the BPSK(1) signal coherent discriminator (infinite bandwidth).

**Figure 10:**
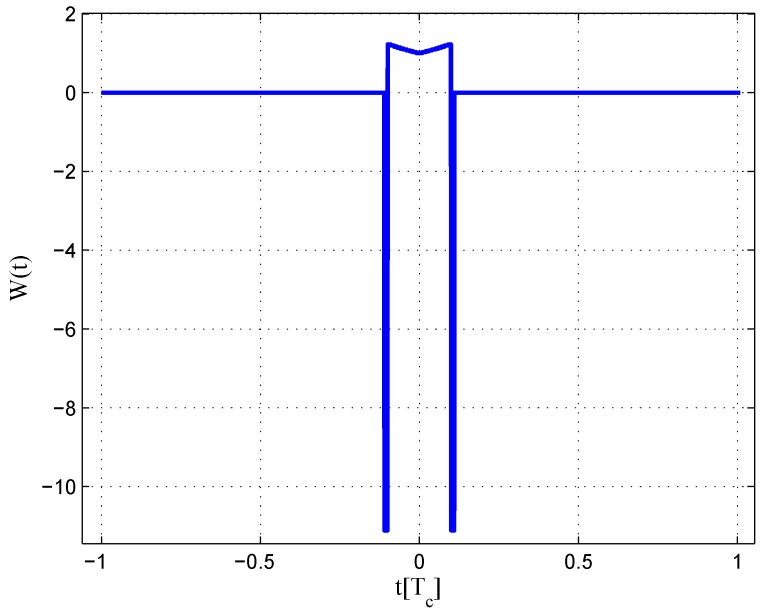
Code correlation reference waveform diagram for the BPSK(1) signal non-coherent discriminator (infinite bandwidth).

**Figure 11:**
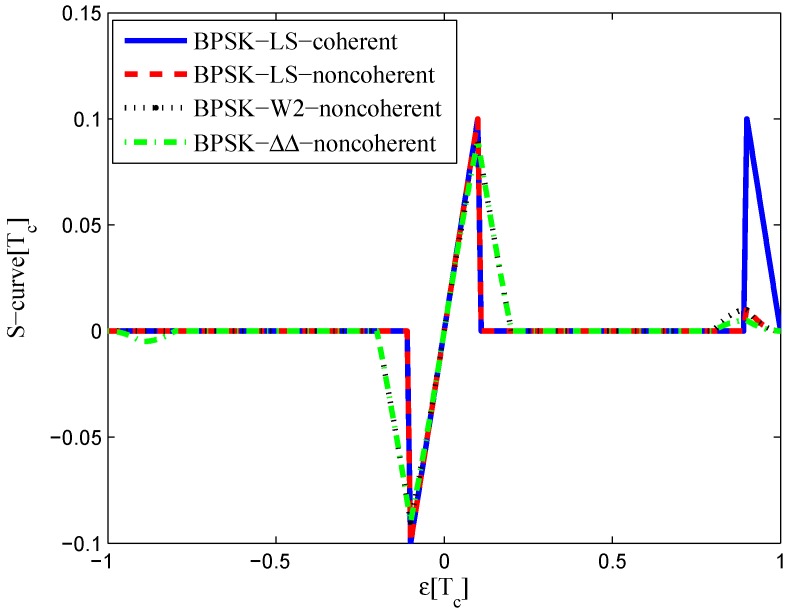
Fitted S-curves for the BPSK(1) signal (infinite bandwidth).

The code correlation reference waveform diagrams for the infinite bandwidth case are shown in Figure 9 and Figure 10. There is little difference between the coherent and non-coherent cases. The coherent waveform is like the W2 waveform, and it presents at every boundary of the original code. The obtained waveform is also formed by four strobes with a symmetrical structure. The linear range of the corresponding S-curve is defined by the width of the center strobes. The strobes from both sides are much narrower, which makes the fitted S-curve quickly vanish to zero. The total area of the coherent waveform is zero. The non-coherent waveform, however, is different in the strobe values, which are not constant. The total area of the non-coherent waveform is also zero. The fitted S-curves for the infinite bandwidth case are presented in Figure 11, compared to the non-coherent W2 and double-delta correlator. Here, we show the S-curve of the coherent discriminator and the S-curve of the non-coherent discriminator. We also show the S-curve of non-coherent W2 and double-delta correlator, which are basically the same. It can be observed that the ideal S-curve is well reproduced by the resulting S-curve. The resulting S-curve returns more quickly to zero than the W2 and double-delta correlator, but slower to the ideal S-curve, which is determined by μ.

**Figure 12:**
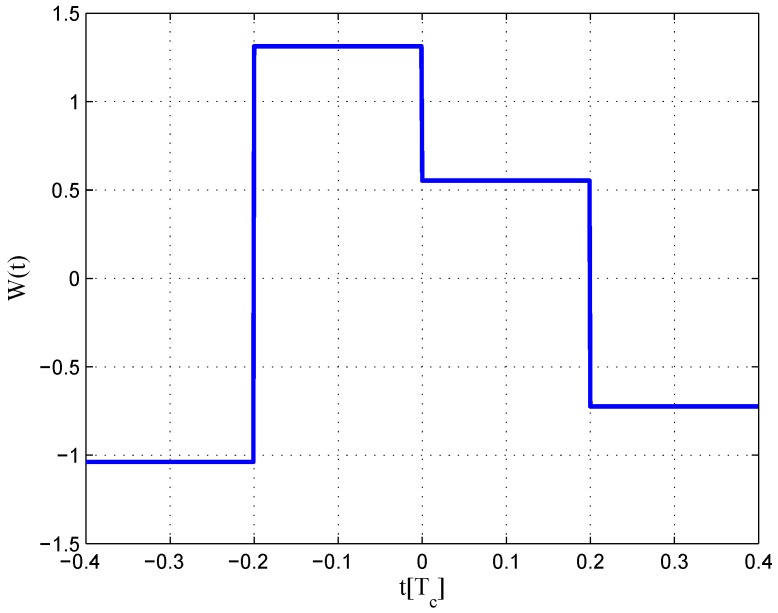
Code correlation reference waveform diagram for the BPSK(1) signal coherent discriminator (4.092-MHz bandwidth).

**Figure 13:**
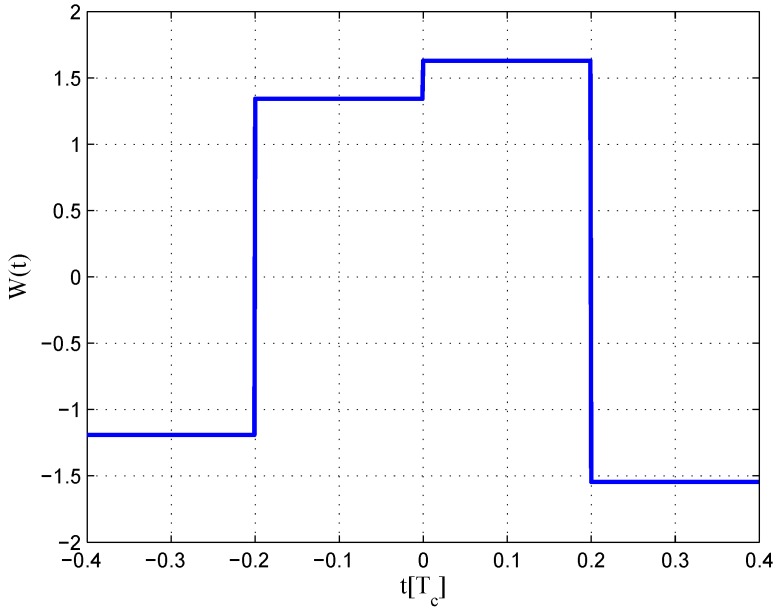
Code correlation reference waveform diagram for the BPSK(1) signal non-coherent discriminator (4.092-MHz bandwidth).

**Figure 14:**
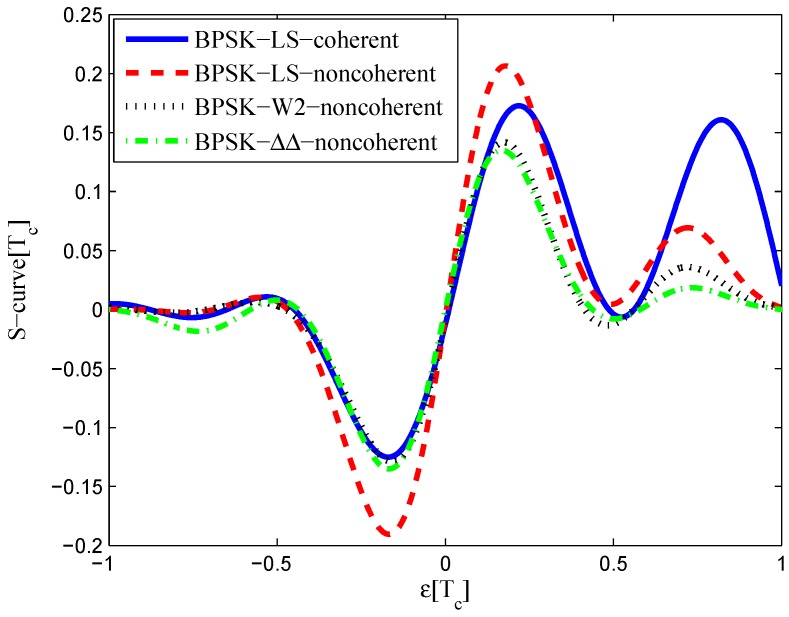
Fitted S-curves for the BPSK(1) signal (4.092-MHz bandwidth).

The results for the 4.092-MHz case are different from the infinite bandwidth case, because we had to increase μ and the size of the linear region. The resulting correlator diagrams for the 4.092-MHz case are shown in Figure 12 and Figure 13. The corresponding S-curves are presented in Figure 14. Again, the resulting S-curve is more similar to the ideal S-curve with higher peak values and a sharper slope in the linear region, compared to W2 and the double-delta correlator. The ideal S-curve is well reproduced by the fitting result, except the nonzero values outside the linear region.

### 3.2. BOC(1,1)

The same procedures as for BPSK signal are repeated for the BOC(1,1) signal. The settings for BOC(1,1) signals are listed in Table 2. However, the type of CCRW in limited bandwidth situations is different from BPSK signals. The code correlation reference waveform for the infinite bandwidth case presents at each code transition, and its value is determined by the sign of the next code at transitions, like the W2 waveform. However, the code correlation reference waveforms for the finite bandwidth cases present at the center of each code, just like BGP waveforms. Its value is determined by the sign of the current code. The waveforms are demonstrated in Figure 4.

**Figure 15:**
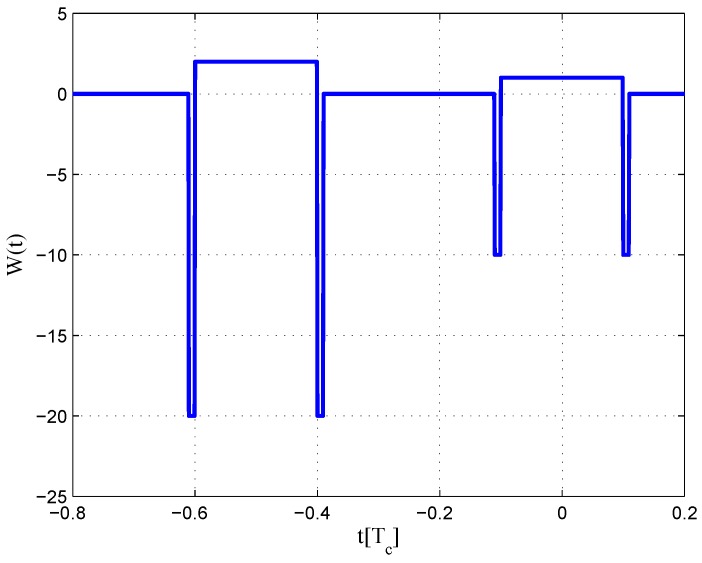
Code correlation reference waveform diagram for the BOC(1,1) signal coherent discriminator (infinite bandwidth).

**Figure 16:**
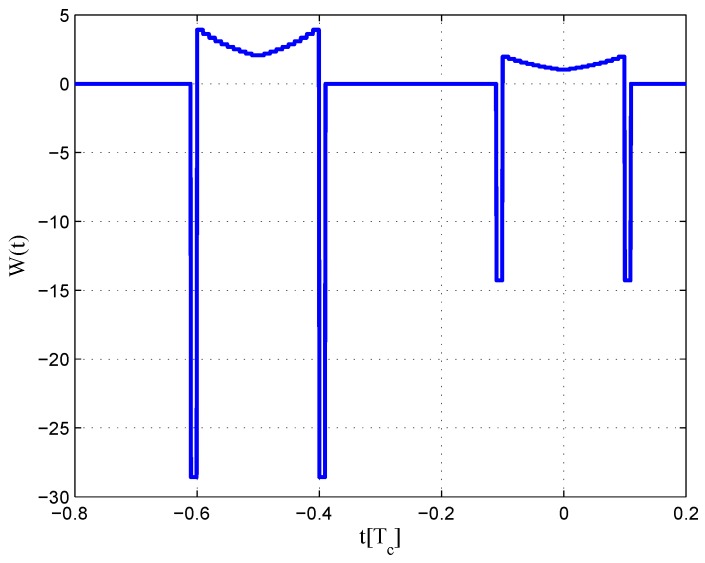
Code correlation reference waveform diagram for the BOC(1,1) signal non-coherent discriminator (infinite bandwidth).

**Figure 17:**
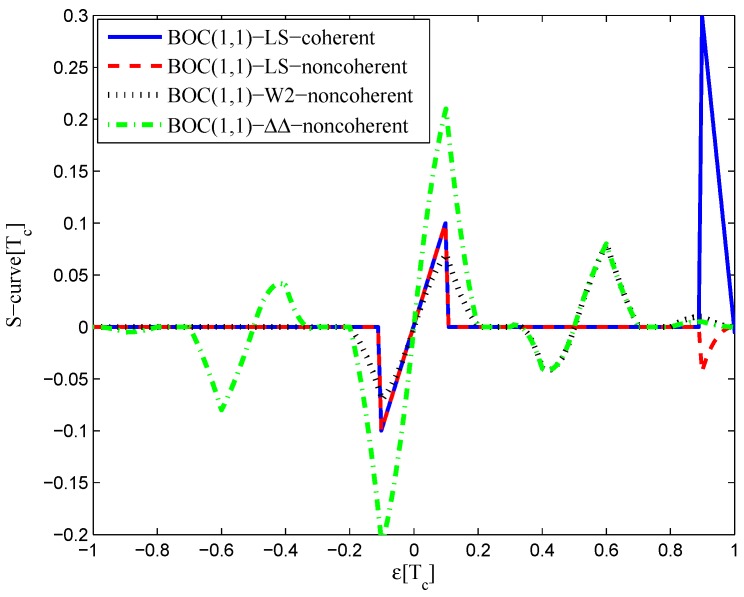
Fitted S-curves for the BOC(1,1) signal (infinite bandwidth).

The code correlation reference waveform diagrams for the infinite bandwidth case are shown in Figure 15 and Figure 16. The structures are quite similar to the BPSK signal with infinite bandwidth, except that an additional waveform appears at the position of −0.5 chips with twice the amplitudes. The resulting S-curves for the infinite bandwidth case are presented in Figure 17, compared to non-coherent W2 and the double-delta correlator. The target S-curve is better reproduced by the coherent and non-coherent LS CCRWs than W2 and the double-delta correlator. Furthermore, they have no false lock points when W2 and the double-delta correlator have one at 0.5 chips.

**Figure 18:**
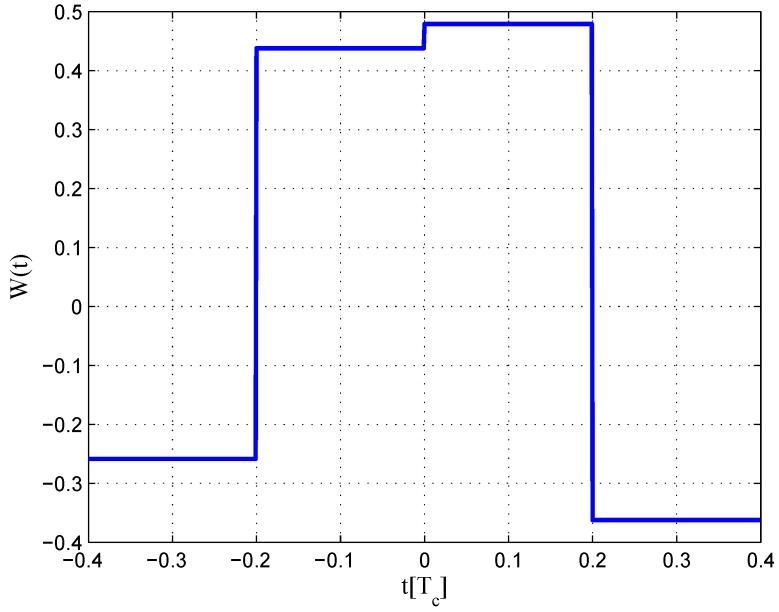
Code correlation reference waveform diagram for the BOC(1,1) signal coherent discriminator (6.138-MHz bandwidth).

**Figure 19:**
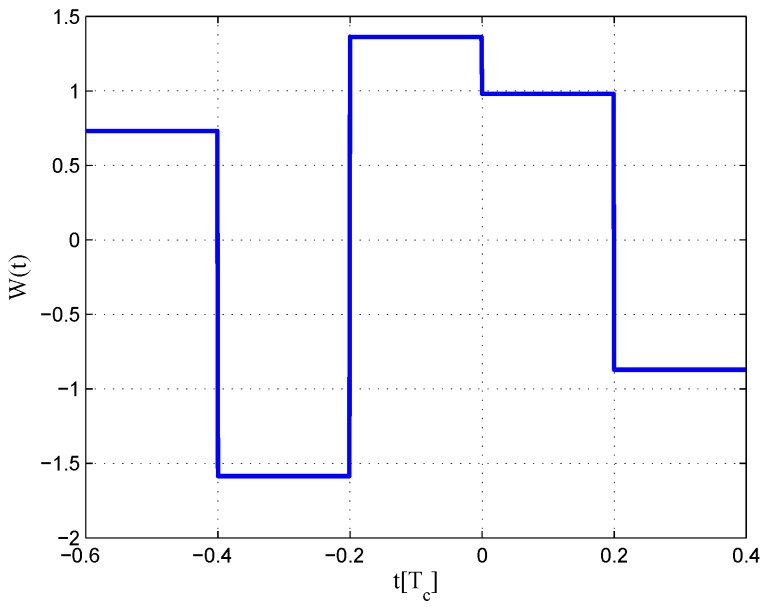
Code correlation reference waveform diagram for the BOC(1,1) signal non-coherent discriminator (6.138-MHz bandwidth).

**Figure 20:**
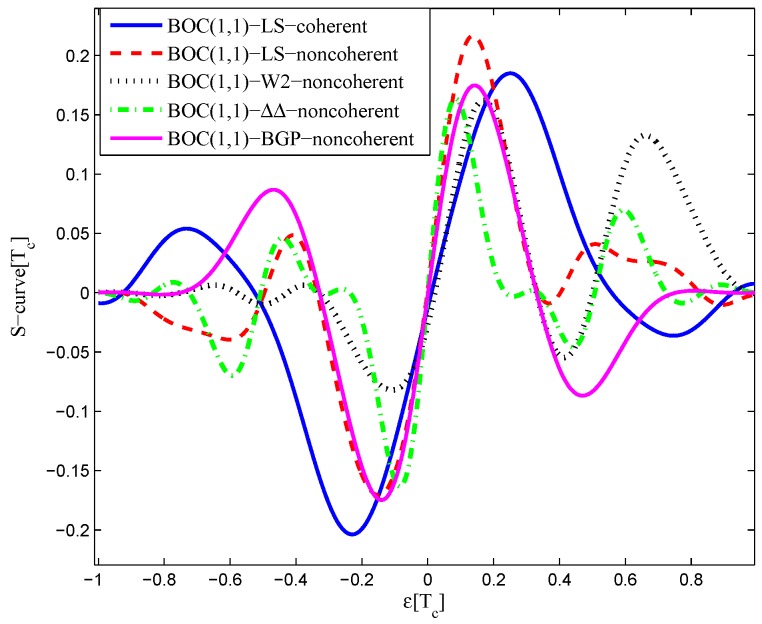
Fitted S-curves for the BOC(1,1) signal (6.138-MHz bandwidth).

The results for the 6.138-MHz bandwidth case are shown in Figure 18, Figure 19 and Figure 20. The results of the BGP waveforms are added in the simulation, since the generated waveform has a similar structure to it. The distribution of waveforms was increased to −0.4–0.4 chips for the coherent case and −0.6–0.4 chips for the non-coherent case. Because we had to increase μ and the size of the linear region due to the limited bandwidth, the resulting S-curves have greater peak values and a sharper slope in the linear region. Unlike W2 and the double-delta correlator, the fitted S-curves do not have a false lock point in the position of 0.5 chips. However, the offset in the ideal S-curve leads to larger fluctuations in the S-curve outside the linear region than W2 CCRW, which would increase the multipath error envelope.

**Figure 21:**
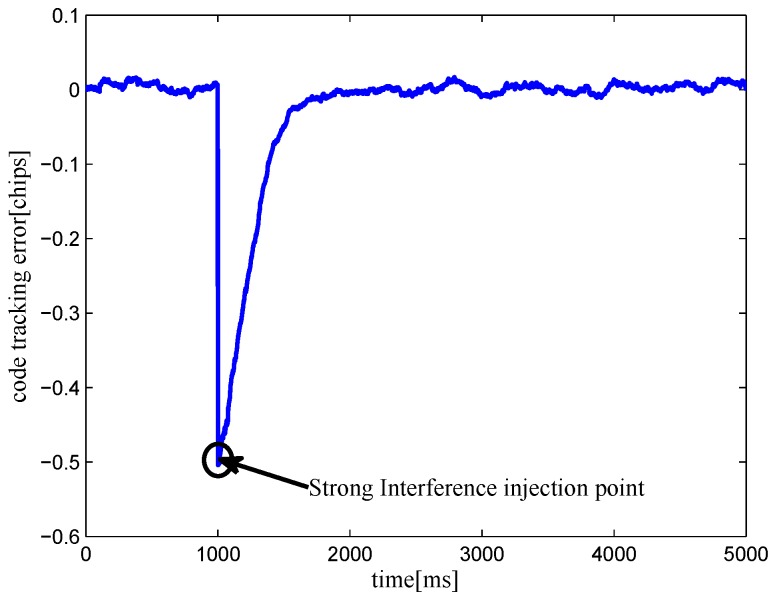
Simulation results of the false lock problem when the LS non-coherent discriminator is applied in a noisy environment (input $C/N_{0}$ = 35 dBHz, injecting a short period of strong interference at a time interval of 1 s, double-sided bandwidth = 6.138 MHz).

The same simulation of Figure 2 was performed for the proposed non-coherent discriminator of the BOC(1,1) signal in Figure 21. This shows the tracking error result returned to the correct positions, because the false lock point is avoided in the S-curve design.

### 3.3. BOC(2,1)

The settings for BOC(2,1) signals are listed in Table 3. The code correlation reference waveform for each case also present at each code transition, and its value is determined by the sign of the next code at transitions.

**Figure 22:**
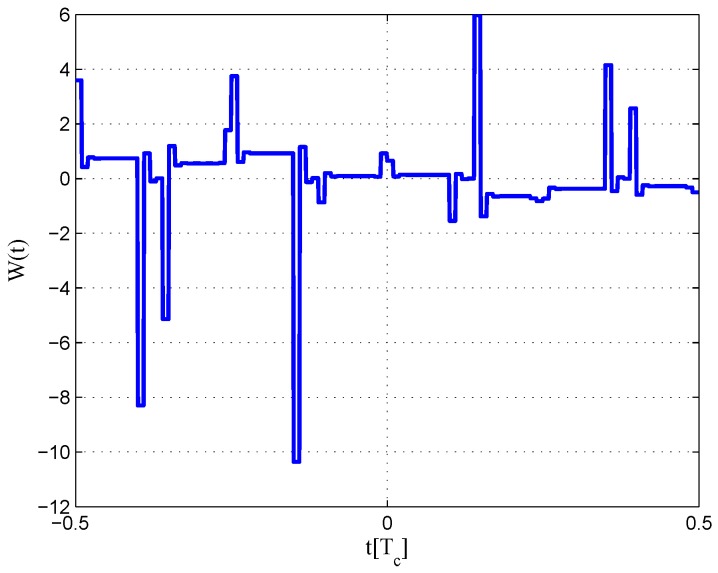
Code correlation reference waveform diagram for the BOC(2,1) signal coherent discriminator (infinite bandwidth).

**Figure 23:**
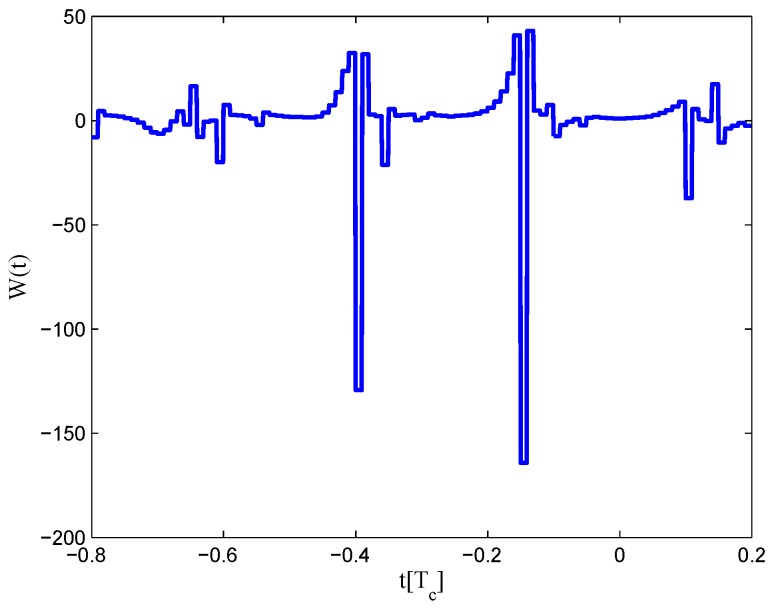
Code correlation reference waveform diagram for the BOC(2,1) signal non-coherent discriminator (infinite bandwidth).

**Figure 24:**
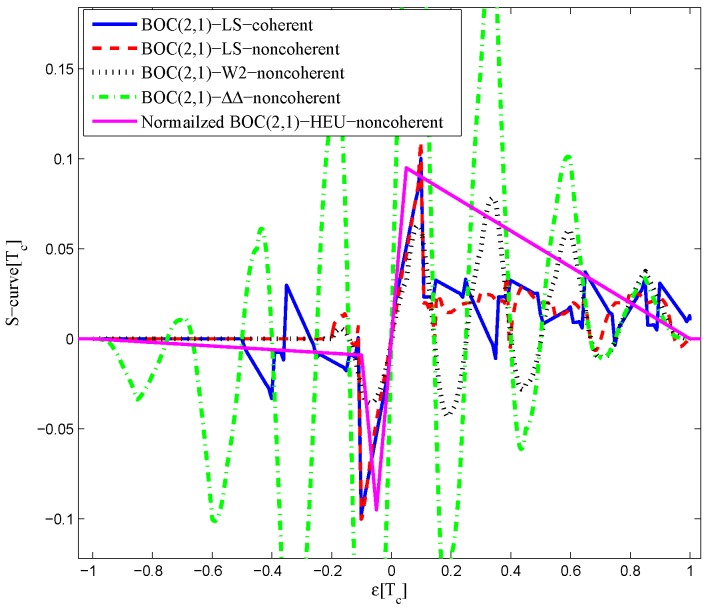
Fitted S-curves for the BOC(2,1) signal (infinite bandwidth).

The CCRW diagrams of the BOC(2,1) signal for the infinite bandwidth case are shown in Figure 22 and Figure 23. The structures are quite different from the BPSK and BOC(1,1) signal with infinite bandwidth. No regular patterns can be found for the BOC(2,1) signals. The resulting S-curves for the infinite bandwidth case are presented in Figure 24, compared to non-coherent W2 CCRW, the double-delta correlator and the results of the waveforms in [14]. It can be seen that the S-curve in [14] (named the HEU S-curve) has a half-size linear region with a doubled slope, compared to the others. The steeper slope in the linear region aims for the improvement of tracking precision. The S-curve proposed here has a slope and a linear region like the W2 waveform, and an offset in the right side and returns to zero in the left side. As a result, the LS S-curves have a better multipath mitigation capability then the Harbin Engineering University (HEU) S-curve for medium and long delayed multipath signals, but worse for the short delayed multipath signal. The HEU S-curve also has a wider pull-in region for the negative part.

**Figure 25:**
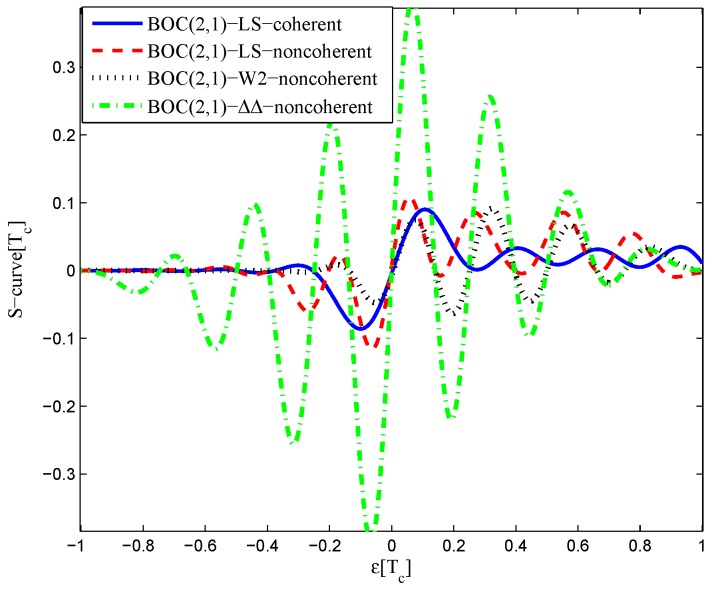
Fitted S-curves for the BOC(2,1) signal (8.184-MHz bandwidth).

**Figure 26:**
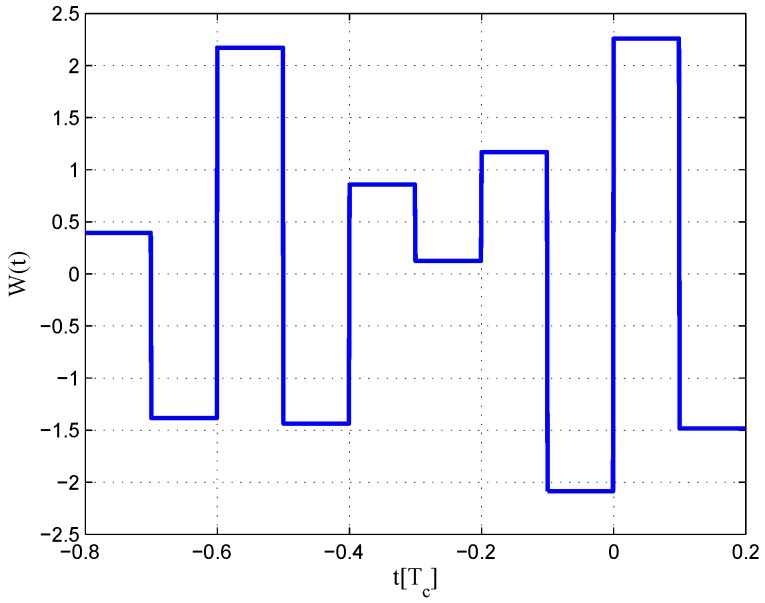
Code correlation reference waveform diagram for the BOC(2,1) signal coherent discriminator (8.184-MHz bandwidth).

**Figure 27:**
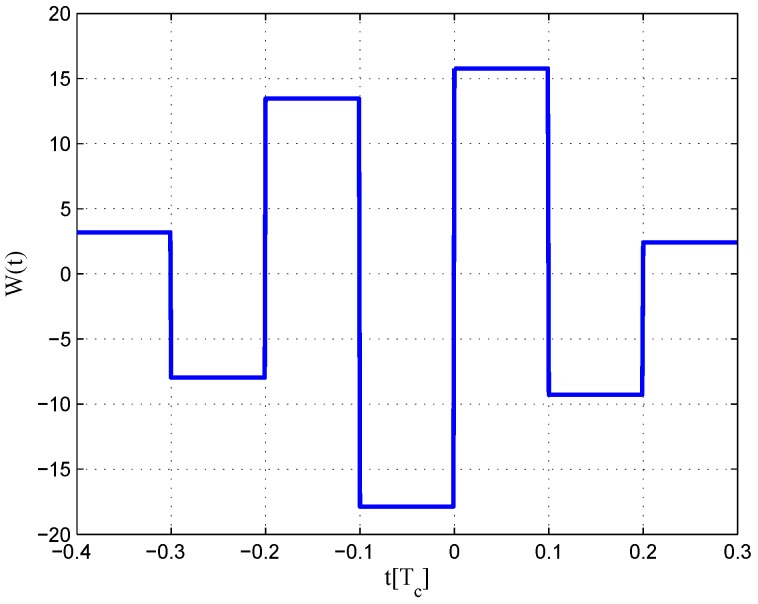
Code correlation reference waveform diagram for the BOC(2,1) signal non-coherent discriminator (8.184-MHz bandwidth).

The resulting S-curves for the 8.184-MHz bandwidth case are presented in Figure 25, and the CCRW diagrams are shown in Figure 26 and Figure 27. The gate width of the code correlation reference waveforms is 0.1 chips, indicating that the smaller bandwidth can increase μ. The ideal S-curve can be reproduced in the linear region, but the non-coherent S-curve has a sharper slope due to the ACF of the BOC(2,1) signal. Furthermore, the non-coherent S-curve returns to zero more quickly and has larger fluctuations in the left side of the linear region. The fitted S-curves still avoid the false lock points, compared to CCRW W2 and the double-delta correlator.

### 3.4. BOC(6,1)

The settings for BOC(6,1) signals are listed in Table 4. The code correlation reference waveform type is the same as the W2 waveform. The least-squares process is performed only for the coherent discriminator case, because the realizable linear region of the given non-coherent discriminator is rather small due to the multi-peaked ACF of the BOC(6,1) signal.

**Figure 28:**
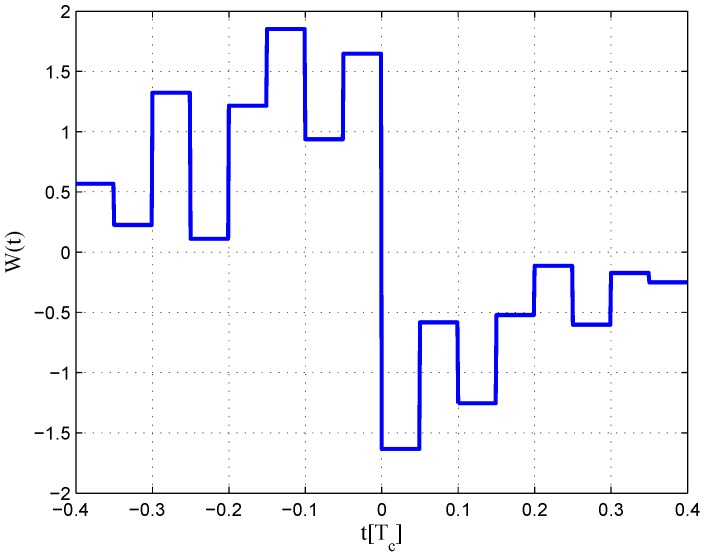
Code correlation reference waveform diagram for the BOC(6,1) signal coherent discriminator (24.552-MHz bandwidth).

**Figure 29:**
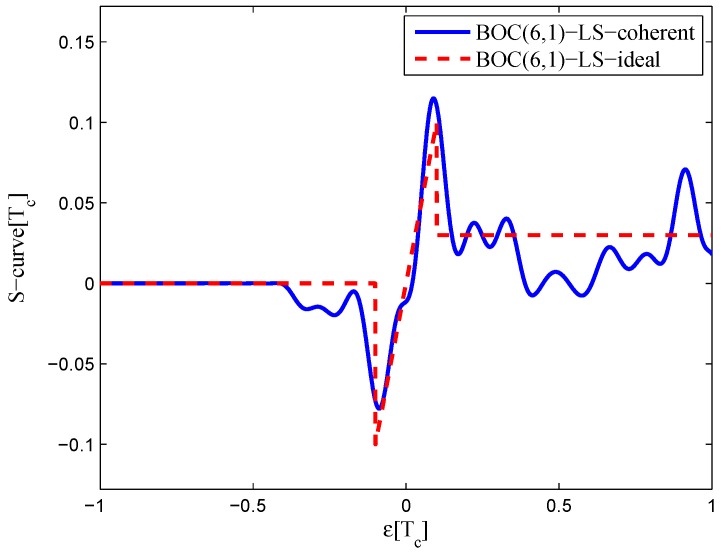
Fitted S-curves for the BOC(6,1) signal (24.552-MHz bandwidth).

The code correlation reference waveform diagram for the 24.552-MHz bandwidth case is shown in Figure 28 and Figure 29. The strobe width is 0.05 chips, and the waveforms are distributed from −0.4–0.4 chips. The resulting S-curves can roughly follow the ideal S-curve, but the peak in the left side of the linear region is reduced to balance the linear regression error. There is also no false lock points in the fitted result.

### 3.5. BOC(7,1)

Similar result are obtained with BOC(7,1) signals. The fitting parameters are listed in Table 5.

**Figure 30:**
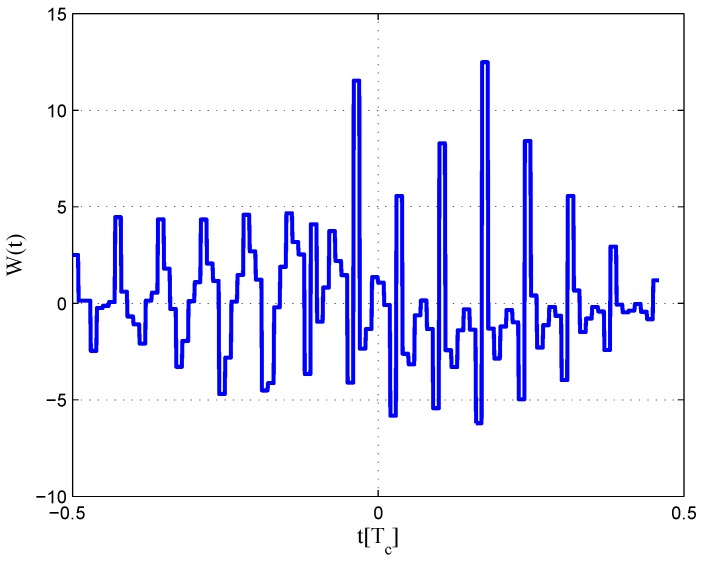
Code correlation reference waveform diagram for the BOC(7,1) signal coherent discriminator (20.46-MHz bandwidth).

For high-order BOC signals, the limited bandwidth and multi-peaked auto-correlation function cause a shift in the zero-crossing point of the fitted S-curve. The code correlation reference waveform diagrams of the BOC(7,1) signal for the 20.46-MHz bandwidth are shown in Figure 30. It is more difficult to find a suitable reference waveform for high-order BOC signals with a larger bandwidth, which is the opposite of the BOC(1,1) signal.

**Figure 31:**
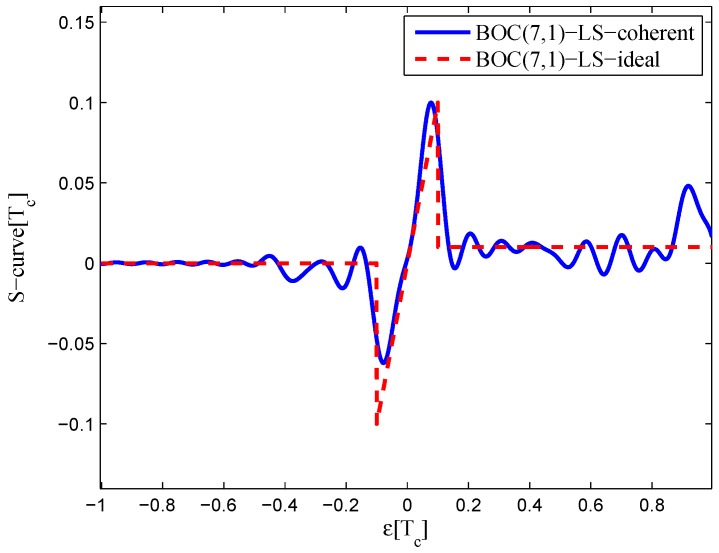
Fitted S-curves for the BOC(7,1) signal (20.46-MHz bandwidth).

The resulting S-curves for the 20.46-MHz bandwidth case are shown in Figure 31. Like the BOC(6,1) signal, the resulting S-curves can avoid the false lock points. However, the peak in the left side of linear region is reduced, and it is difficult to restrain the fluctuations outside the linear region.

## 4. Performance Analysis

There are some evaluation criteria for the GNSS code tracking discriminators, like sensitivity, precision, stability, etc. Here, the multipath error envelope and the tracking jitter of the proposed discriminators are evaluated, since they are mostly concerned during tracking. The multipath envelopes of these discriminators are analyzed and compared to the CCRW W2 and the double-delta correlator.

### 4.1. Multipath Error Envelope

The signal multipath error envelope is defined as the minimum and maximum code tracking errors caused by the multipath interference as a function of the multipath delay, when only one multipath signal exists [15]. The amplitude of the multipath signal is often assumed to be half of the amplitude of the direct signal. The multipath error envelope is usually plotted within one chip, because the multipath delays larger than one chip are rare and their errors usually negligibly small.

#### 4.1.1. BPSK(1)

**Figure 32:**
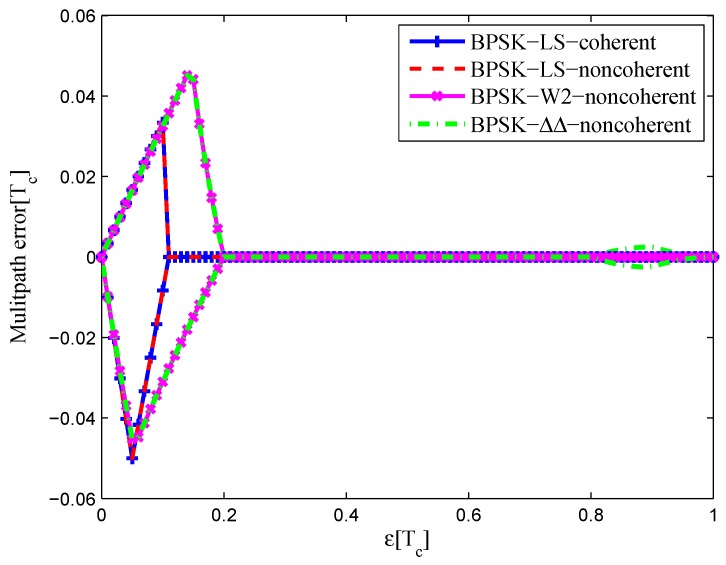
Multipath error envelopes for S-curves of the BPSK(1) signal (infinite bandwidth).

Figure 32 shows the multipath error envelope for the BPSK(1) signal with infinite bandwidth. Comparisons are made between the coherent discriminator of the LS method, the non-coherent discriminator of the LS method, the discriminator of W2 and the discriminator of the double-delta correlator. It can been seen that the two discriminators of the LS method have almost the same area. Furthermore, the multipath error envelope of CCRW W2 is about the same as the envelope of the double-delta correlator, while it does not have an embossment in the 0.9 chips. The area of each multipath error envelope is shown in Table 6, the coherent LS discriminator has a minimum envelope area, which is 95.6% of the non-coherent LS discriminator envelope, 47.3% of the W2 envelope and 44.9% of the double-delta correlator envelope.

**Figure 33:**
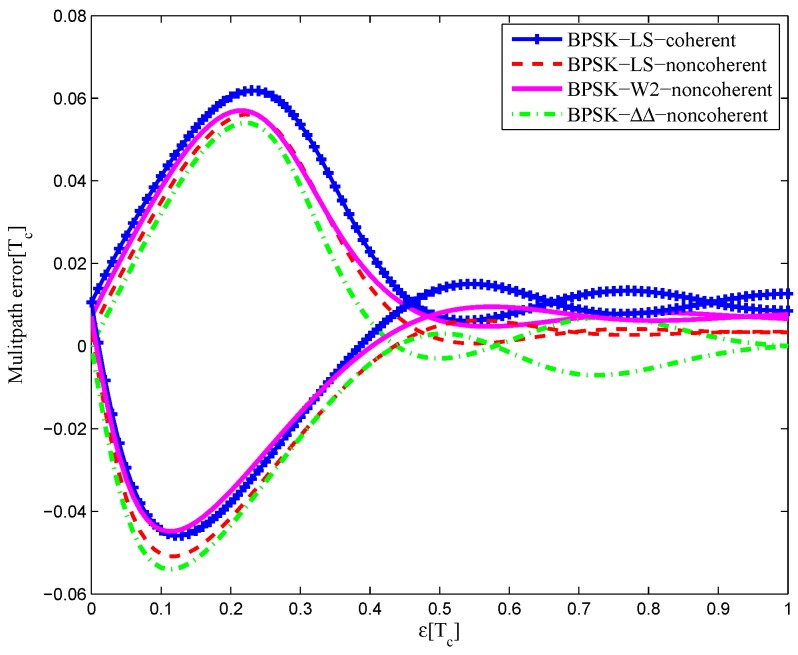
Multipath error envelopes for the S-curves of the BPSK(1) signal (4.092-MHz bandwidth).

The multipath error envelops of the BPSK(1) signal with the 4.092-MHz bandwidth are shown in Figure 33. Each discriminator has a zero-crossing point offset at different levels. The LS coherent discriminator has a zero-crossing point shift of 0.01 chips, and the CCRW W2 discriminator has a shift of about 0.075 chips. However, the zero-crossing points of the other two discriminators are not affected by the limited bandwidth. In all, the coherent LS discriminator has a minimum envelope area, which is 88.2% of the non-coherent LS discriminator envelope, 92.7% of the W2 envelope and 83.8% of the double-delta correlator envelope.

#### 4.1.2. BOC(1,1)

**Figure 34:**
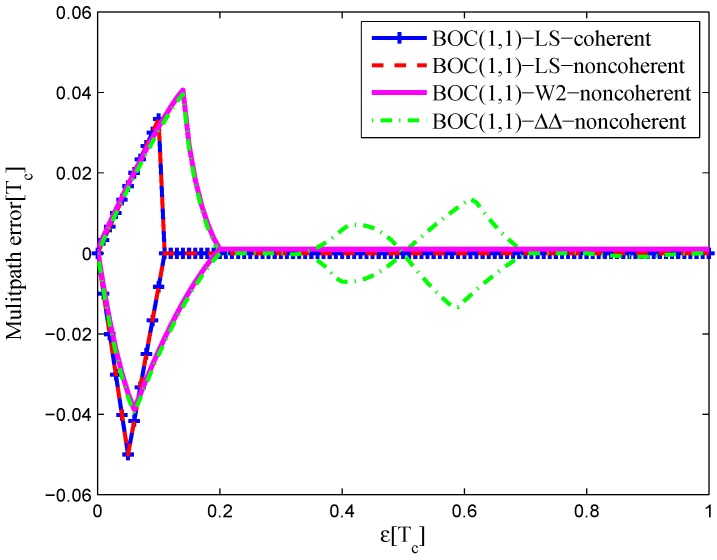
Multipath error envelopes for the S-curves of the BOC(1,1) signal (infinite bandwidth).

The area of BOC(1,1) signal multipath error envelope is shown in Table 7. Figure 34 shows the multipath error envelope for the BOC(1,1) signal of infinite bandwidth. It is interesting that the envelopes of the coherent and non-coherent discriminator of the LS method are the same, like the BPSK(1) signal case. The W2 and double-delta correlator multipath error envelope are about the same, as well, but the double-delta correlator has two additional side lobes at 0.5 chips due to the false lock points at that position. In all, the coherent LS discriminator has a minimum envelope area, which is the same as the non-coherent LS discriminator envelope, 60.5% of the W2 envelope and 38.7% of the double-delta correlator envelope.

**Figure 35:**
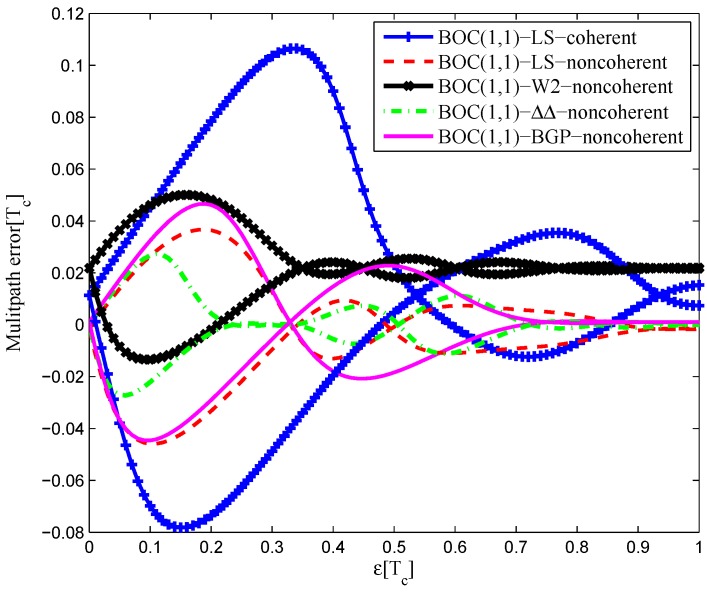
Multipath error envelopes for the S-curves of the BOC(1,1) signal (6.138-MHz bandwidth).

The multipath error envelopes of the BOC(1,1) signal with the 6.138-MHz bandwidth are shown in Figure 35. The CCRW W2 discriminator has a zero-crossing point offset of 0.02 chips. The double-delta correlator has a minimum envelope area. The LS discriminators have side lobes at the position of 0.5 chips, due to the fluctuations of the S-curves in the left side to the linear region. However, the non-coherent discriminator still has a smaller area of the multipath error envelope than the BGP discriminator. In all, the double-delta correlator discriminator has a minimum envelope area, which is 17.0% of the coherent LS discriminator envelope, 46.8% of the non-coherent LS discriminator envelope, 75.7% of the W2 envelope and 41.1% of the BGP envelope.

#### 4.1.3. BOC(2,1)

**Figure 36:**
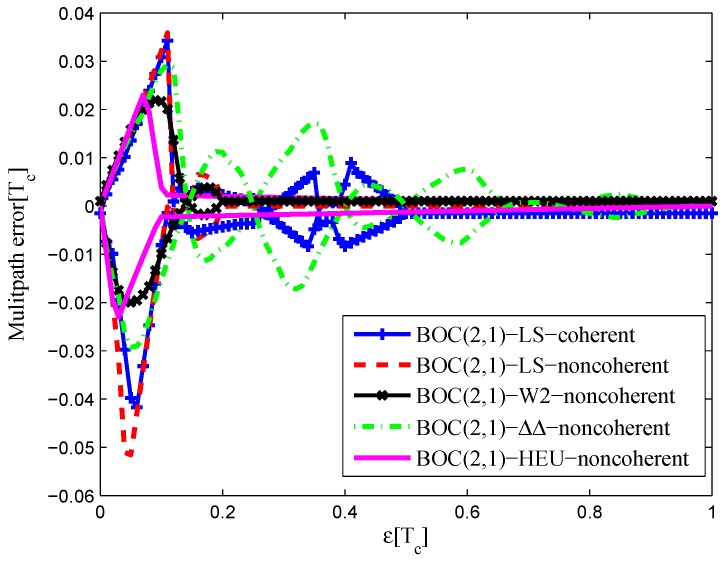
Multipath error envelopes for the S-curves of the BOC(2,1) signal (infinite bandwidth).

Figure 36 shows the multipath error envelope for the BOC(2,1) signal of infinite bandwidth. The LS non-coherent discriminator and W2 have a comparable multipath error envelope because their S-curve is flat in the left side to the linear region. The HEU S-curve has a smaller MEE, because of the narrower operation range. However, the proposed LS S-curve here appears to be much better at mitigating multipath effects where there are medium and long multipath delays, as a result of the narrower negative pull-in region.

The area of each multipath error envelope is shown in Table 8. In all, the W2 discriminator has a minimum envelope area, which is 68.4% of the coherent LS discriminator envelope, 68.4% of the non-coherent LS discriminator envelope, 33.1% of the double-delta correlator envelope and 86.7% of the HEU discriminator envelope.

**Figure 37:**
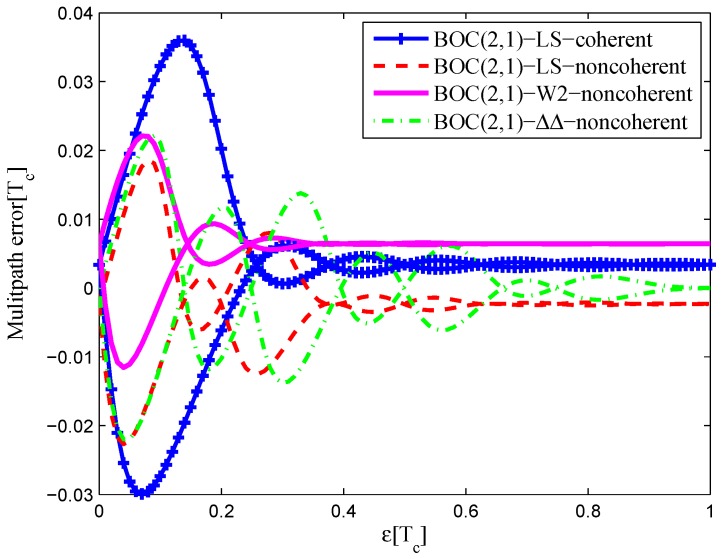
Multipath error envelopes for the S-curves of the BOC(2,1) signal (8.184-MHz bandwidth).

A similar conclusion can be drawn from the 8.184-MHz bandwidth case in Figure 37. The CCRW W2 discriminator is shifted in the zero-crossing point about 0.005 chips, but it still has a minimum envelope. The LS coherent and non-coherent discriminators have a shift about −0.005 chips. In all, the CCRW W2 discriminator has a minimum envelope area, which is 32.4% of the coherent LS discriminator envelope, 57.6% of the non-coherent LS discriminator envelope, 34.6% of the double-delta correlator envelope and 60.7% of the HEU discriminator envelope.

#### 4.1.4. BOC(6,1) and BOC(7,1)

**Figure 38:**
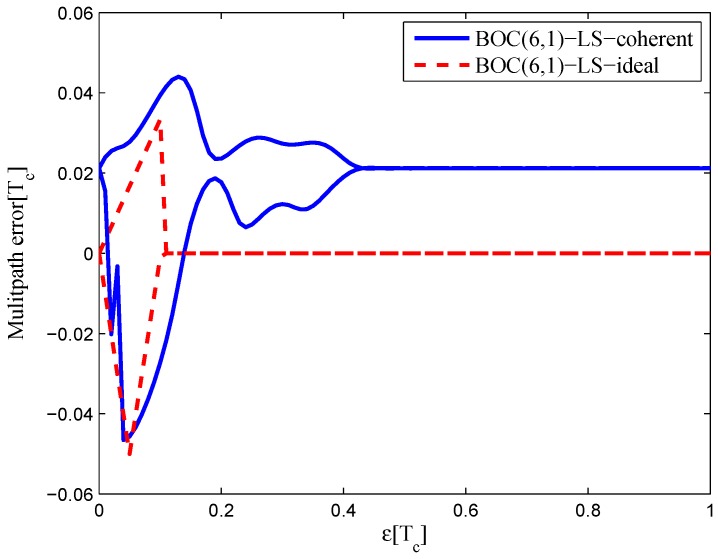
Multipath error envelopes for the S-curves of the BOC(6,1) signal (24.552-MHz bandwidth).

Figure 38 shows the multipath error envelope for the BOC(6,1) signal of the 24.552-MHz bandwidth, compared to the multipath error envelope of the ideal S-curve. The LS coherent discriminator has a zero-crossing point offset of 0.02 chips because of the imperfect fitting.

**Figure 39:**
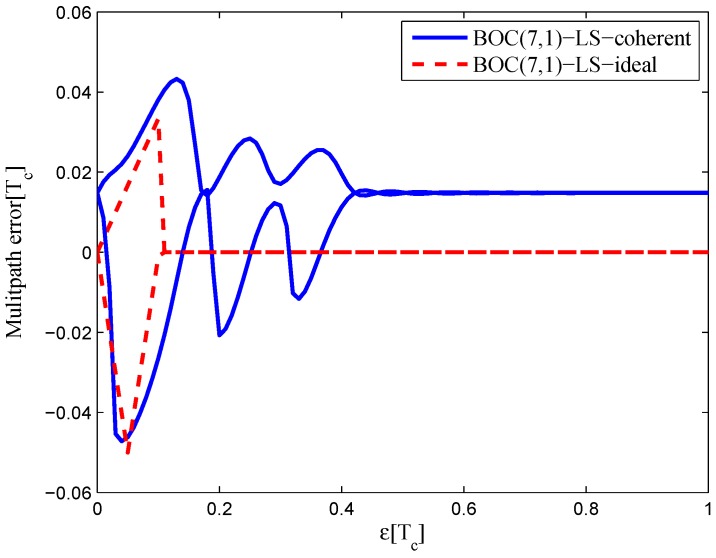
Multipath error envelopes for the S-curves of the BOC(7,1) signal (20.46-MHz bandwidth).

Figure 39 shows the multipath error envelope for the BOC(7,1) signal of the 20.46-MHz bandwidth. The results are similar to the BOC(6,1) signal. The LS coherent discriminator has a zero-crossing point offset of −0.005 chips. The area of each multipath error envelope for BOC(6,1) and BOC(7,1) signals is shown in Table 9.

In all, the LS coherent discriminator outperforms other techniques in the area of MEE for the BPSK signal and the BOC(1,1) signal with infinite bandwidth. The LS non-coherent discriminator has a similar performance as the W2 technique for the BPSK signal. For band-limited BOC(1,1) and BOC(2,1) signals, the W2 discriminator has the least MEE area.

### 4.2. Tracking Jitter

The tracking jitter [9] quantifies the amount of noise transferred by a tracking loop from the input signal to the final delay estimate. If the thermal noise is only considered, the output of the non-coherent discriminator is given by:

(14) $$\begin{array}{ccc} d_{nc}\left(\varepsilon \right) & = & \left(\sqrt{2C}R_{\tilde{X}X}\left(\varepsilon \right)\cos\left(\varphi_{e}\right)+N_{IX}\right)\left(\sqrt{2C}R_{\tilde{X}W}\left(\varepsilon \right)\cos\left(\varphi_{e}\right)+N_{I}\right)\\ & + & \left(\sqrt{2C}R_{\tilde{X}X}\left(\varepsilon \right)\sin\left(\varphi_{e}\right)+N_{QX}\right)\left(\sqrt{2C}R_{\tilde{X}W}\left(\varepsilon \right)\sin\left(\varphi_{e}\right)+N_{Q}\right) \end{array}$$

Here, *C* stands for the signal power. $N_{IX}$ and $N_{QX}$ are the noise items of the in-phase and quadrature-phase branch during the prompt code correlation, with the variance of $R_{XX}\left(0\right)N_{0}/T$. When the phase lock loop (PLL) is stable, $\varphi_{e}\approx 0$. Then, the discriminator can be simplified as follows.

(15) $$\begin{array}{ccc} d_{nc}\left(\varepsilon \right) & = & 2CR_{\tilde{X}X}\left(\varepsilon \right)R_{\tilde{X}W}\left(\varepsilon \right)+\sqrt{2C}R_{\tilde{X}W}\left(\varepsilon \right)N_{IX}\\ & + & \sqrt{2C}R_{\tilde{X}X}\left(\varepsilon \right)N_{I}+N_{IX}N_{I}+N_{QX}N_{Q} \end{array}$$

Using the linear model at ε=0 for the code discriminator, it can be shown that the discriminator is as follows.

(16) $$\begin{array}{c} d_{nc}\left(\varepsilon \right)=g_{0}\varepsilon +N \end{array}$$

Here, $g_{0}$ represents the gain of the discriminator, and *N* means the Gaussian white noise with zero mean. Then, Equation (16) can be transformed into Equation (17).

(17) $$\begin{array}{ccc} g_{0} & = & 2CR_{\tilde{X}X}\left(0\right)R_{\tilde{X}W}^{\prime }\left(0\right)\\ N & = & \sqrt{2C}R_{\tilde{X}X}\left(0\right)N_{I}+\sqrt{2C}R_{\tilde{X}W}\left(0\right)N_{IX}+N_{IX}N_{I}+N_{QX}N_{Q} \end{array}$$

Here, we assume that the channel impulse response is undistorted and that the correlation function of the received signal between the local replica is symmetric, i.e., $R_{\tilde{X}X}^{\prime }\left(0\right)=0$. Furthermore, the designed reference waveform W(t) ensures $R_{\tilde{X}W}\left(0\right)=0$. The variance of *N* can be calculated as follows.

(18) $$\begin{array}{ccc} \sigma_{N}^{2} & = & E[N^{2}]\\ & = & 2CR_{\tilde{X}X}^{2}\left(0\right)E\left[N_{I}^{2}\right]+2CR_{\tilde{X}W}^{2}\left(0\right)E\left[N_{IX}^{2}\right]+E\left[N_{IX}^{2}N_{I}^{2}\right]+E\left[N_{QX}^{2}N_{Q}^{2}\right]\\ & = & 2CR_{\tilde{X}X}^{2}\left(0\right)R_{WW}\left(0\right)N_{0}/T+2R_{XX}\left(0\right)R_{WW}\left(0\right){\left(N_{0}/T\right)}^{2} \end{array}$$

From the above, the steady state normalized code error variance is given by [16]:

(19) $$\begin{array}{c} \sigma_{N}^{2}=\frac{2B_{L}T\sigma_{N}^{2}}{g_{0}^{2}}=\frac{B_{L}RWW\left(0\right)}{R_{\tilde{X}W}^{\prime 2}\left(0\right)C/N_{0}}\left[1+\frac{1}{R_{\tilde{X}X}^{2}\left(0\right)C/N_{0}T}\right] \end{array}$$

The output of coherent discriminator is given by:

(20) $$\begin{array}{ccc} d_{c}\left(\varepsilon \right) & = & \left(\sqrt{2C}R_{\tilde{X}X}\left(\varepsilon \right)\cos\left(\varphi_{e}\right)+N_{IX}\right)\\ & + & \left(\sqrt{2C}R_{\tilde{X}X}\left(\varepsilon \right)\sin\left(\varphi_{e}\right)+N_{QX}\right) \end{array}$$

Similarly, the corresponding steady state normalized code error variance can be given by:

(21) $$\begin{array}{c} \sigma_{N}^{2}=\frac{2B_{L}R_{WW}\left(0\right)}{R_{\tilde{X}W}^{\prime 2}\left(0\right)C/N_{0}} \end{array}$$

In this case, the tracking jitters of the proposed discriminators are compared to that of the W2 and double-delta technique, assuming $B_{L}=1$ Hz and T=1 ms.

#### 4.2.1. BPSK(1)

**Figure 40:**
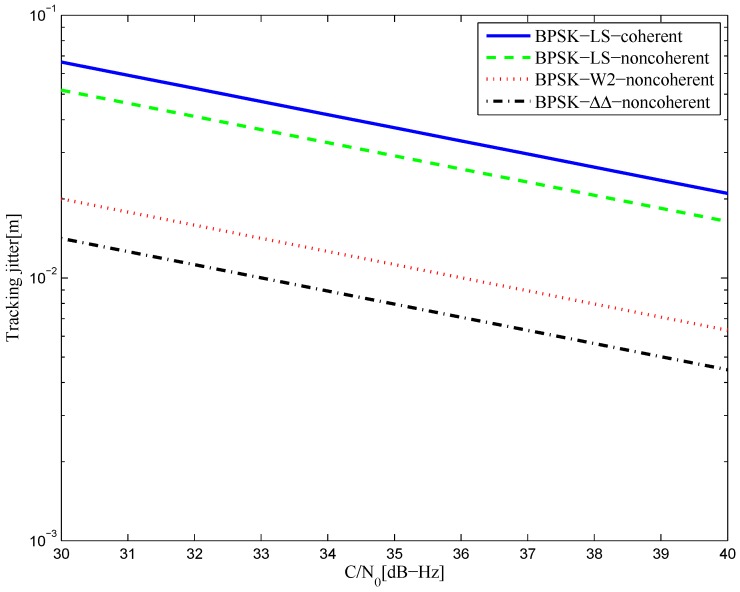
The tracking jitters for the S-curves of the BPSK(1) signal (infinite bandwidth).

**Figure 41:**
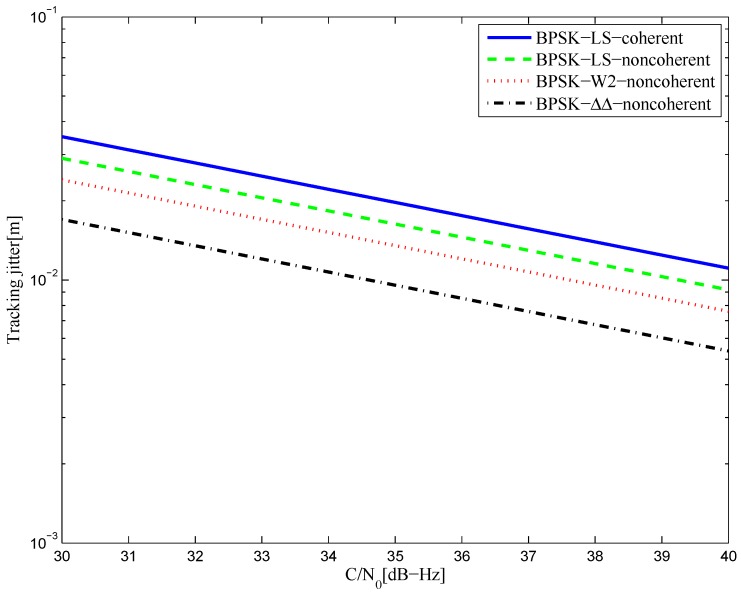
The tracking jitters for the S-curves of the BPSK(1) signal (4.092-MHz bandwidth).

Figure 40 shows the tracking jitters of simulated S-curve of BPSK(1) signal with infinite bandwidth. The coherent and non-coherent S-curves are worse than that of the W2 and double-delta technique. This is mainly due to the greater amplitudes of their code reference waveforms, which decide the value of $R_{WW}\left(0\right)$, when there is little difference in $R_{\tilde{X}W}^{\prime }\left(0\right)$ of different waveforms. Specifically, the CCRW W2 and double-delta correlator waveforms constitute strobes with amplitudes of one and −1, while there is no amplitude constraint on the LS reference waveforms. The $R_{WW}\left(0\right)$ of the double-delta correlator is half the value of W2’s, since the double-delta correlator waveform occurs at the transitions of each code, while the W2 waveform occurs at the edge of each code. Therefore, the double-delta correlator discriminator outperforms the W2 discriminator. Similar results are shown in Figure 41 for the 4.092-MHz bandwidth case.

#### 4.2.2. BOC(1,1)

**Figure 42:**
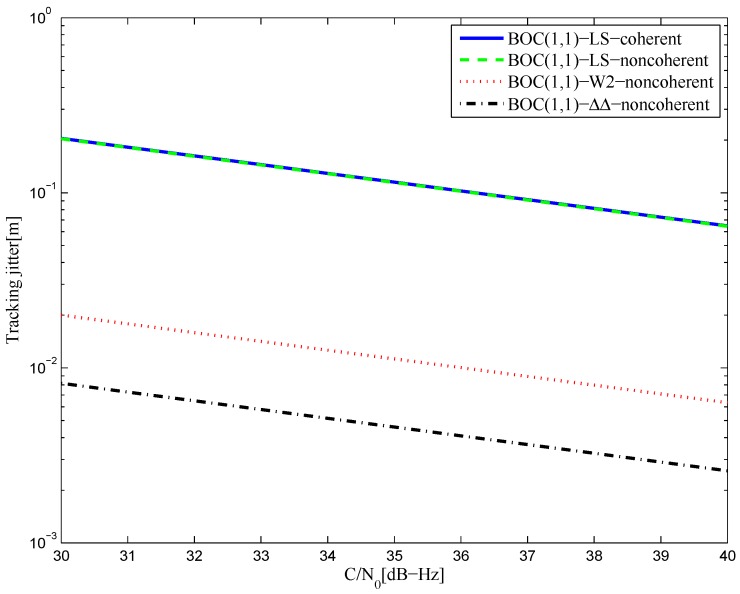
The tracking jitters for the S-curves of the BOC(1,1) signal (infinite bandwidth).

**Figure 43:**
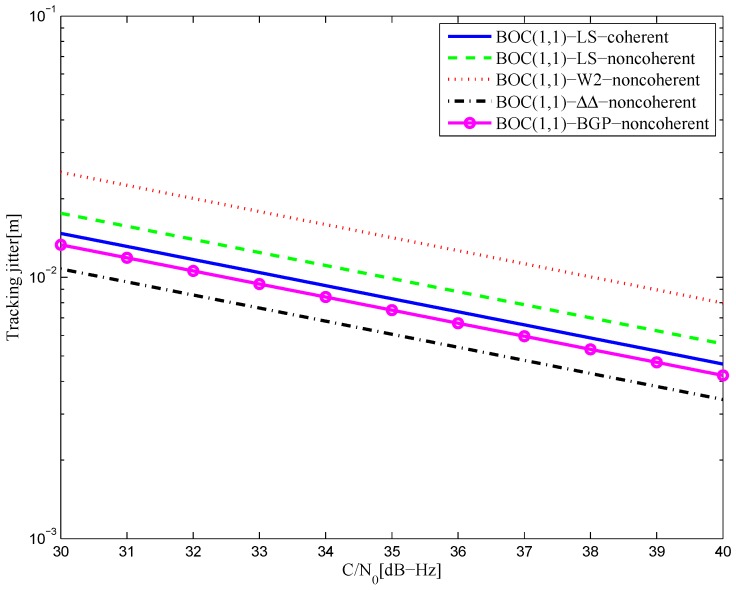
The tracking jitters for the S-curves of the BOC(1,1) signal (6.138-MHz bandwidth).

Figure 42 shows the tracking jitters of simulated S-curves for the BOC(1,1) signal of infinite bandwidth. The performance of the LS coherent S-curve discriminator is basically the same as the LS non-coherent S-curve, but both are still worse than that of the CCRW W2 discriminator and the double-delta correlator discriminator. The reason is the same as for the BPSK signal. The results of the 6.138-MHz bandwidth case are shown in Figure 43. The performances of the fitted S-curves are even better than the W2 discriminator. This implies that the LS method can keep its performance for the band-limited situation, whereas the W2 discriminator experiences the worst loss.

#### 4.2.3. BOC(2,1)

**Figure 44:**
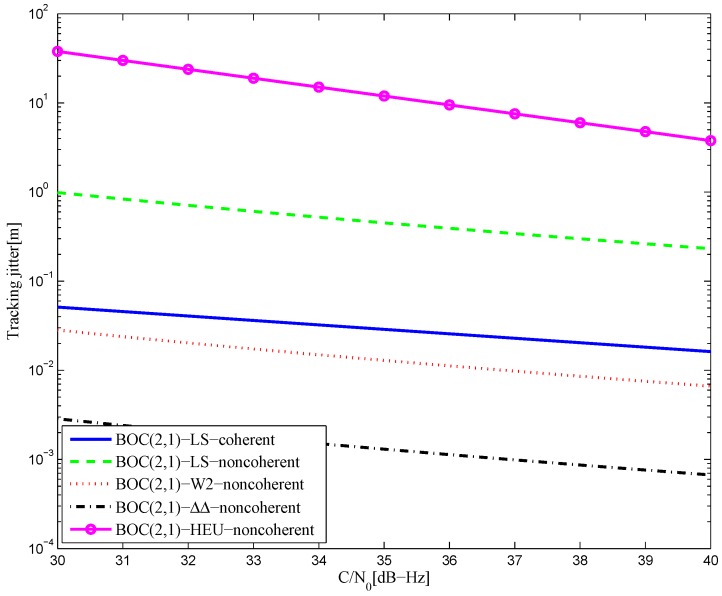
The tracking jitters for the S-curves of the BOC(2,1) signal (infinite bandwidth).

**Figure 45:**
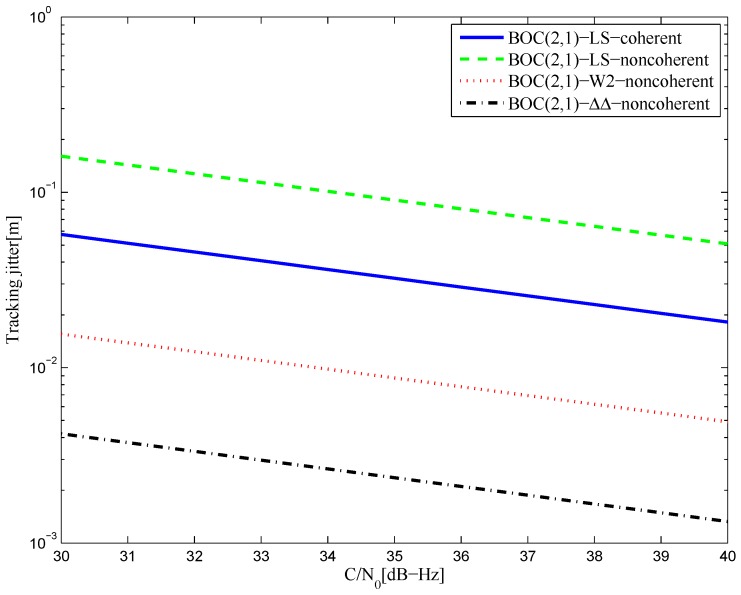
The tracking jitters for the S-curves of the BOC(2,1) signal (8.184-MHz bandwidth).

Figure 44 shows the tracking jitters of simulated S-curves for the BOC(2,1) signal of infinite bandwidth, which is similar to the BPSK signal case. The results of the 8.184-MHz bandwidth case are shown in Figure 45. The code tracking error variance of the proposed S-curve is much better than the HEU S-curve, because of the closely-spaced correlator positions and large correlator weights in the [14] discriminator. By contrast, the amplitudes of the local reference waveform in our manuscript are relatively low, and it has a narrower distribution.

#### 4.2.4. BOC(6,1) and BOC(7,1)

**Figure 46:**
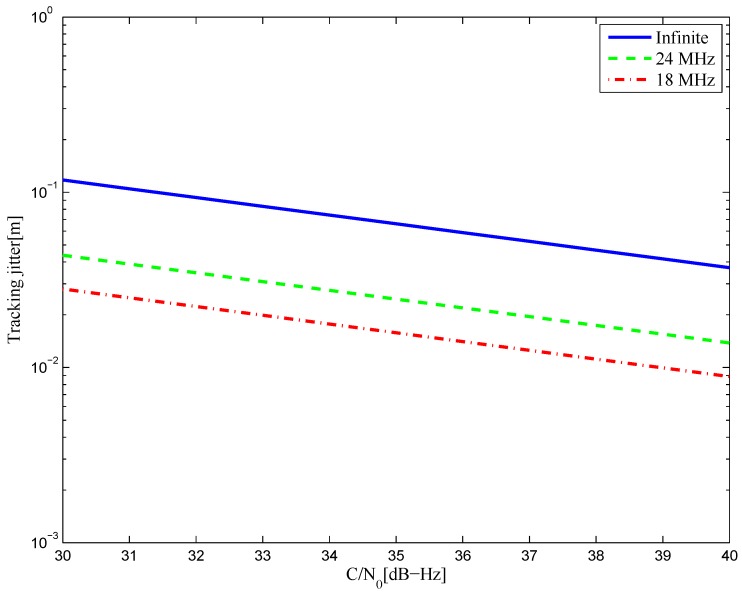
The tracking jitters for the S-curves of the BOC(6,1) signal.

**Figure 47:**
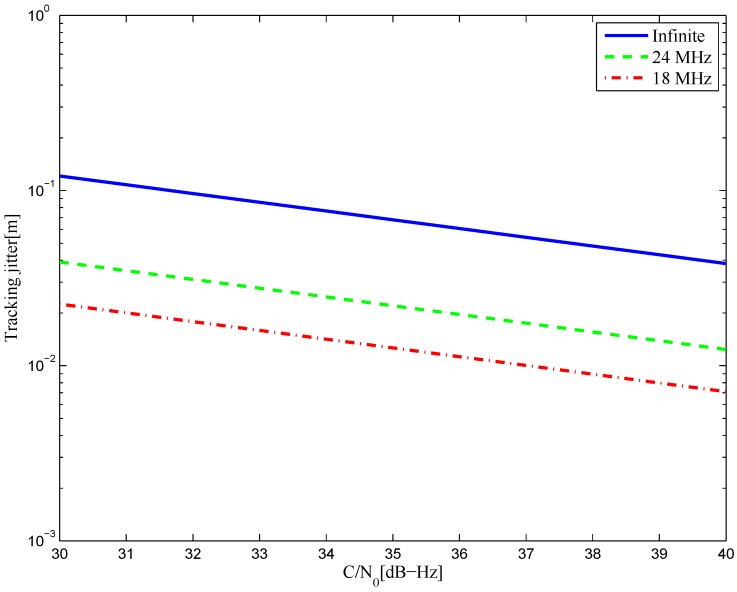
The tracking jitters for the S-curves of the BOC(7,1) signal.

The tracking jitters of the resulting S-curves for the BOC(6,1) signal are shown in Figure 46. It is shown that better results are acquired with a smaller bandwidth, which implies that the amplitude of the reference waveform is generally smaller with a narrower bandwidth. Further, this means that the resulting S-curve for the narrower bandwidth case is more easily realized. The simulation result is shown in Figure 47 for the BOC(7,1) signal.

In all, the W2 non-coherent discriminator outperforms the simulated LS discriminators in the tracking jitters, except for the case of the BOC(1,1) signal with the 6.138-MHz bandwidth. This is because the complexity of LS waveforms has impacted the tracking precision, which is the same for the HEU S-curve.

## 5. Discussion

Firstly, the simulation results proved the realizability of the proposed method, given the accordance between the resulting S-curves and target S-curves. Apparently, better consistency can be acquired if the strobe width is smaller and the waveform length is larger. However, a smaller strobe width means a higher sampling frequency in the receiver, which would increase the hardware cost. Therefore, the results in the infinite bandwidth cases are not practical, as it demands a sampling frequency above 100 MHz. However, it shows the capability of the LS method in finding suitable CCRWs with a given pre-correlation bandwidth and strobe width. For example, the acquired LS coherent CCRW for the BPSK signal infinite bandwidth case is similar to the W2 waveform, which is designed theoretically. Furthermore, the non-coherent CCRW of the BPSK signal with infinite bandwidth produces an S-curve more similar to the ideal S-curve than the non-coherent W2 discriminator.

Secondly, the optimization process of the LS method is a balance between the multipath error envelope, linear region length and the false lock points. The multipath error envelope of the S-curve is determined by the left side of the linear region, while the false lock points occur at the right side of the linear region. Thus, the whole area of the S-curve is constrained during the approximation. A close confinement on one aspect may deteriorate another aspect, e.g., a strictly restricted S-curve for the smallest MEE area could lead to the very existence of false lock points. Therefore, the resulting non-coherent S-curve without false lock points usually has larger MEE than W2, which is designed without considering the false lock points. However, the multipath mitigation capability of LS coherent discriminators is better than W2 for the BPSK signal. Furthermore, they are all capable of resisting the multipath signals with medium or large time delays and perform worse with multipath signals arriving within 0.1 chips than the direct signal.

Thirdly, the ideal S-curve may not be the best approximation target, because the restrictions of the right side to the linear region can be relaxed to be just above zero, rather than a positive constant. In a similar manner, this relaxed restriction of the S-curve can also avoid false lock points. However, this vague description is difficult to realize in the LS process and can be studied in future research. The reason to relax the restriction is that it is difficult to find a perfect reference waveform that meets the demands of being a non-coherent discriminator with a small MEE area, high tracking precision, no false lock points and narrow bandwidth simultaneously, especially for the high-order BOC signals. Thus, a relaxed restriction may improve the performance.

## 6. Conclusions

An LS technique is proposed for the design of CCRW for BPSK and BOC signals in GNSS, using the truncated SVD method. The purpose of this technique is to find an appropriate receiving discriminator for unambiguous and anti-multipath tracking in band-limited situations. The results show that this technique is capable of generating relatively simple local reference waveforms for coherent and non-coherent discriminators in different bandwidth cases, except that the BOC(6,1) and BOC(7,1) only have coherent results. The corresponding S-curves are capable of avoiding the false lock points. Their multipath error envelopes of the coherent discriminator are about 60% of the W2 CCRW for the BPSK signal, about twice for the BOC(1,1) signal and about three times for the BOC(2,1) signal. Their multipath error envelopes of the non-coherent discriminator are about the same as the W2 CCRW for the BPSK signal, except that it is half of W2 CCRW with infinite bandwidth, about 150% for the BOC(1,1) signal and about twice for the BOC(2,1) signal. The standard tracking errors of acquired S-curves are usually larger than W2 CCRW, except for the cases of the BOC(1,1) signal. Therefore, the LS method can acquire S-curves without false lock points at the cost of MEE area and tracking precision.

## Figures and Tables

**Table 1:** Simulation parameters for the BPSK(1) signal.

Scenario	Infinite Bandwidth	16.368 MHz	8.184 MHz	4.092 MHz
Fitting range (±chips)	1	1	1	1
Linear region (±chips)	0.1	0.1	0.1	0.2
Offset (chips)	0	0.01	0.01	0.01
Waveform range (chips)	−0.11∼0.11	−0.15∼0.15	−0.2∼0.2	−0.4∼0.4
Gate width (chips)	0.01	0.05	0.1	0.2

**Table 2:** Simulation parameters for the BOC(1,1) signal.

Scenario	Infinite Bandwidth	24.552 MHz	12.276 MHz	6.138 MHz
Fitting range (±chips)	1	1	1	1
Linear region (±chips)	0.1	0.1	0.1	0.2
Offset (chips)	0	0.02	0.03	0.03
Waveform range (chips)	−0.11∼0.11	−0.15∼0.15	−0.2∼0.2	−0.4∼0.4
Gate width (chips)	0.01	0.05	0.1	0.2

**Table 3:** Simulation parameters for the BOC(2,1) signal.

Scenario	Infinite Bandwidth	16.368 MHz	8.184 MHz
Fitting range (±chips)	1	1	1
Linear region (±chips)	0.1	0.1	0.1
Offset (chips)	0 (coherent)	0.02 (coherent)	0.02 (coherent)
	0.02 (noncoherent)	0.03 (noncoherent)	0.05 (noncoherent)
Waveform range (chips)	−0.5∼0.5 (coherent)	−0.8∼0.15 (coherent)	−0.8∼0.2 (coherent)
	−0.8∼0.2 (noncoherent)	−0.5∼0.5 (noncoherent)	−0.4∼0.4 (noncoherent)
Gate width (chips)	0.01	0.05 (coherent), 0.02 (noncoherent)	0.1

**Table 4:** Simulation parameters for the BOC(6,1) signal.

Scenario	Infinite Bandwidth	24.552 MHz	18.414 MHz
Fitting range (±chips)	1	1	1
Linear region (±chips)	0.1	0.1	0.1
Offset (chips)	0.01	0.03	0.03
Waveform range (chips)	−0.5∼0.5	−0.4∼0.4	−0.4∼0.4
Gate width (chips)	0.01	0.05	0.05

**Table 5:** Simulation parameters for the BOC(7,1) signal.

Scenario	Infinite Bandwidth	20.46 MHz	16.368 MHz
Fitting range (±chips)	1	1	1
Linear region (±chips)	0.1	0.1	0.1
Offset (chips)	0.01	0.03	0.02
Waveform range (chips)	−0.5∼0.5	−0.6∼0.4	−0.3∼0.3
Gate width (chips)	0.01	0.05	0.05

**Table 6:** The multipath error envelope of the BPSK(1) signal.

Bandwidth	Coherent	Non-Coherent	W2	ΔΔ
Infinite (chip${}^{2}$)	0.0044	0.0046	0.0093	0.0098
16.368 MHz (chip${}^{2}$)	0.0066	0.0098	0.0097	0.0103
8.184 MHz (chip${}^{2}$)	0.0067	0.0086	0.0082	0.0088
4.092 MHz (chip${}^{2}$)	0.0253	0.0287	0.0273	0.0302

**Table 7:** The multipath error envelope of the BOC(1,1) signal.

Bandwidth	Coherent	Non-Coherent	W2	ΔΔ	BGP
Infinite (chip${}^{2}$)	0.0046	0.0046	0.0076	0.0119	
24.552 MHz (chip${}^{2}$)	0.0219	0.0140	0.0081	0.0129	0.0181
12.27 M6Hz (chip${}^{2}$)	0.0192	0.0086	0.0060	0.0095	0.0157
6.138 MHz (chip${}^{2}$)	0.0676	0.0246	0.0152	0.0115	0.0280

**Table 8:** The multipath error envelope of the BOC(2,1) signal.

Bandwidth	Coherent	Non-Coherent	W2	ΔΔ	HEU
Infinite (chip${}^{2}$)	0.0057	0.0057	0.0039	0.0118	0.0045
16.368 MHz (chip${}^{2}$)	0.0143	0.0120	0.0047	0.0144	0.0039
8.184 MHz (chip${}^{2}$)	0.0105	0.0059	0.0034	0.0103	0.0056

**Table 9:** The multipath error envelope of the BOC(6,1) and BOC(7,1) signals.

Bandwidth	BOC(6,1)	BOC(7,1)
Infinite (chip${}^{2}$)	0.0108	0.0087
24.552 MHz (chip${}^{2}$)	0.0119	
20.46 MHz (chip${}^{2}$)		0.0093
18.414 MHz (chip${}^{2}$)	0.0119	
16.368 MHz (chip${}^{2}$)		0.0093

## Abbreviations

The following abbreviations are used in this manuscript:

GNSS	Global Navigation Satellite System
CCRW	Code correlation reference waveform
BOC	Binary offset carrier
BPSK	Binary phase shift keying
LS	Least-squares
SVD	Singular value decomposition
BGP	BOC-gated-PRN
PRS	Public Regulated Service
CNR	Carrier-to-noise ratio
GPS	Global Positioning System
GLONASS	Global Navigation Satellite System
CCF	Cross-correlation function
ACF	Auto-correlation function
PLL	Phase lock loop
DLL	Delay lock loop
MEE	Multipath error envelope
PRN	Pseudo-random noise
PSD	Power spectrum density
HEU	Harbin Engineering University
